# Multi-line Adaptive Perimetry (MAP): A New Procedure for Quantifying Visual Field Integrity for Rapid Assessment of Macular Diseases

**DOI:** 10.1167/tvst.7.5.28

**Published:** 2018-10-16

**Authors:** Steven M. Thurman, Marcello Maniglia, Pinakin G. Davey, Mandy K. Biles, Kristina M. Visscher, Aaron R. Seitz

**Affiliations:** 1 U.S. Army Research Laboratory, Human Research and Engineering Directorate, Aberdeen Proving Ground, MD, USA; 2 Department of Psychology, University of California, Riverside, Riverside, CA, USA; 3 College of Optometry, Western University, Pomona, CA, USA; 4 Department of Psychology, University of Alabama at Birmingham, Birmingham, AL, USA

**Keywords:** retina, macular disease, microperimetry, metamorphopsia, psychophysics

## Abstract

**Purpose:**

In order to monitor visual defects associated with macular degeneration (MD), we present a new psychophysical assessment called multiline adaptive perimetry (MAP) that measures visual field integrity by simultaneously estimating regions associated with perceptual distortions (metamorphopsia) and visual sensitivity loss (scotoma).

**Methods:**

We first ran simulations of MAP with a computerized model of a human observer to determine optimal test design characteristics. In experiment 1, predictions of the model were assessed by simulating metamorphopsia with an eye-tracking device with 20 healthy vision participants. In experiment 2, eight patients (16 eyes) with macular disease completed two MAP assessments separated by about 12 weeks, while a subset (10 eyes) also completed repeated Macular Integrity Assessment (MAIA) microperimetry and Amsler grid exams.

**Results:**

Results revealed strong repeatability of MAP and high accuracy, sensitivity, and specificity (0.89, 0.81, and 0.90, respectively) in classifying patient eyes with severe visual impairment. We also found a significant relationship in terms of the spatial patterns of performance across visual field loci derived from MAP and MAIA microperimetry. However, there was a lack of correspondence between MAP and subjective Amsler grid reports in isolating perceptually distorted regions.

**Conclusions:**

These results highlight the validity and efficacy of MAP in producing quantitative maps of visual field disturbances, including simultaneous mapping of metamorphopsia and sensitivity impairment.

**Translational Relevance:**

Future work will be needed to assess applicability of this examination for potential early detection of MD symptoms and/or portable assessment on a home device or computer.

## Introduction

Vision is the main modality with which we navigate and interact with the environment. Consequently, visual pathologies can induce limitations that dramatically impair quality of life. The incidence of visual diseases is increasing with the overall aging population, leading to serious public health issues.^1^ Macular degeneration (MD) in particular represents the leading cause of blindness in the Western World^2^ and it is projected to affect 3 million people by 2020 in the United States alone.^3^ MD impairs primarily central vision and has a detrimental impact on daily tasks such as navigating, reading, and recognizing faces, which can impose a severe cost to patients, their families, and the health care system at-large.^4^

An early indicator of MD is the presence of subretinal pockets of amorphous material called drusen, which can cause morphological changes to the retinal array of photoreceptors and result in measurable changes to perception such as regions that appear blurry or distorted.^5^ This type of visual defect, termed metamorphopsia, is a hallmark feature of MD that in some cases may manifest even before disease onset and can correlate tightly with locations of anatomical features from retinal imaging.^6^ Later stage progression of MD involves photoreceptor loss and central scotomata that can cause complete loss of high-acuity central vision.^7^ Currently, there are limited treatment options for MD, particularly in the earliest stages, and no standard intervention exists. Better understanding of the etiology of MD is important for prevention and treatment,^8,9^ but other factors may be crucial for effective management of the progressive disease including (1) a validated procedure for earliest possible detection of disease onset, as well as detecting clinically relevant changes in disease status (e.g., choroidal neovascularization, or CNV), and (2) a viable at-home method to quantitatively track disease progression via self-assessment between visits to the clinic to continuously inform the course of treatment.

The primary protocol for at-home monitoring of visual defects associated with MD is the Amsler grid. In its standard form, the Amsler grid is a self-administered paper-and-pencil assessment that consists of a grid of equally spaced horizontal and vertical lines, similar to a piece of graph paper,^10^ in which users are instructed to fixate the center and subjectively report all locations that appear perturbed or distorted in any way. MD patients often report regions where the straight lines appear blurry, wavy, or missing altogether. While the Amsler grid is ubiquitous due to affordability, portability, and relative ease of use, there are well-documented practical limitations associated with the Amsler grid including poor sensitivity in detecting visual defects, low compliance rates, and limited reproducibility due to the subjective nature of reports.^11^ In fact, the Amsler grid was not designed to produce quantitative measurements of visual defects, nor to distinguish subtle features characterizing the nature of those deficits. Rather, the Amsler is best understood as a coarse diagnostic tool for detecting central vision impairment, but it does not provide reliable information about the precise nature of the underlying disturbance.

Since the degree of perceptual disturbance associated with MD naturally tends to mirror the progression of underlying disease status (i.e., retinal disarray or photoreceptor loss), a fine-grained quantitative assessment of visual field integrity could provide an effective means for tracking MD symptomology to allow earlier intervention, for example, by detecting sudden onset of CNV to immediately refer patients to the clinic, or by monitoring posttreatment remediation of visual function. Recent computerized assessments, such as the Reichter Foresee preferential hyperacuity perimetry (PHP),^12^ the Foresee HOME,^13^ the Radial Shape Discrimination test (RSD),^14^ and the D-chart,^15^ aim to localize and quantify visual field defects using patient responses to carefully controlled visual stimuli and specialized scoring algorithms. For example, PHP presents a horizontal or vertical dotted line in which a subset of elements is slightly misaligned in the shape of a small Gaussian “bump.” The patient is instructed to indicate the location of the distorted region on each trial, and MD patients with pathological distortion will often indicate erroneous locations on the straight part of the line that appear more distorted than the actual target bump. By accumulating data across dozens of trials, the PHP assessment can reference a normative database and estimate the amplitude and likely location of pathological distortions.^16^ A research study showed that PHP had higher sensitivity in detecting CNV in patients with age-related MD in comparison to the Amsler grid,^17^ demonstrating the potential value of using computerized assessments in clinical examination of MD.^18^ However, to date no computerized assessment has been adopted in standard clinical practice to augment the Amsler grid in daily/weekly at-home assessment of vision.

In this paper, we present a new computerized assessment called multiline adaptive perimetry (MAP) that is designed to assess functional integrity of the central visual field by providing quantitative measurements of pathological distortions (e.g., metamorphopsia), as well as more severe defects associated with visual sensitivity loss (e.g., scotoma). The MAP task requires participants to identify bump-like targets embedded in straight dotted lines that cross the central visual field, similar in appearance to stimuli used in the PHP assessment, but it includes several methodological and analytical distinctions including (1) the presentation of multiple simultaneous lines in each trial to facilitate various behavioral responses, which we hypothesized would deliver better time efficiency of the exam, (2) a procedure to sample visual space adaptively on a trial-by-trial basis to hone-in on regions suspected of retinal damage and maximize information gained throughout the assessment, and (3) statistical cluster analysis of three categories of responses (hits, misses, and false alarms [FAs]) to simultaneously parse the visual field into regions that are either functionally healthy, indicative of metamorphopsia, or indicative of more generalized loss of visual sensitivity (e.g., scotoma).

## Methods

### Participants

In experiment 1, we recruited 20 healthy participants (mean age 19.3 years, 10 males) from the UC Riverside Department of Psychology Subject Pool. Participants were given class credit for participation. The protocol was approved by the UC Riverside Institutional Review Board (IRB), and all participants gave written informed consent prior to the study.

In experiment 2, we recruited eight clinical patients diagnosed with various forms of macular disease at the University of Alabama, Birmingham. Visual characteristics, age, and diagnosis of the eight participants are shown in Table 1. Participants were offered transportation and were paid 10 dollars per hour for voluntary participation. The study was approved by the University of Alabama, Birmingham IRB, and all participants gave written informed consent prior to the study. Both studies adhered to the principles set forth in the Declaration of Helsinki.

**Table 1:**
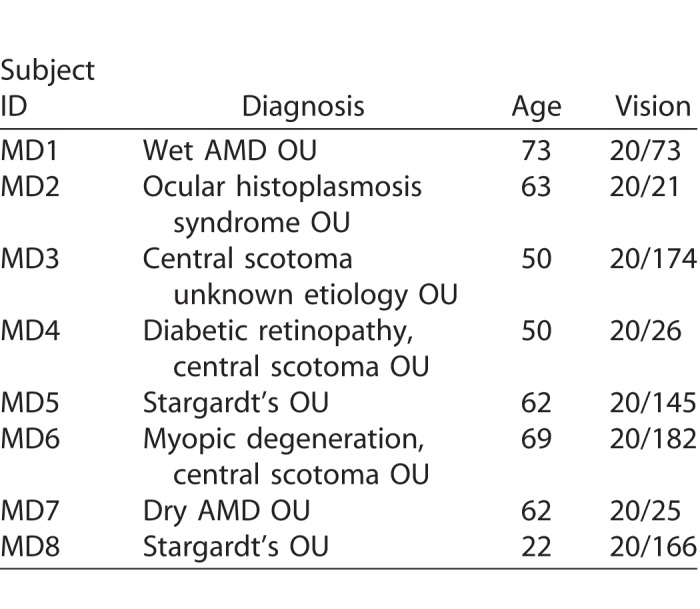
Age, Clinical Diagnosis and Visual Acuity of 8 Patient Participants in Experiment 2

### Method

#### Multiline Adaptive Perimetry (MAP)

**Figure 1:**
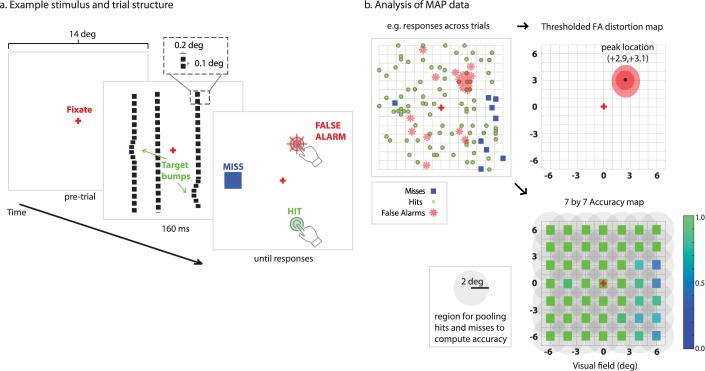
Schematic of the MAP to assess visual field integrity. (a) Upon fixation, horizontal or vertical dotted lines are flashed on the screen for 160 ms (200 ms for MD participants). A variable number of target bumps are introduced in the lines, displacing a subset of elements. Participants indicate where line distortions are perceived, and responses are classified as hits, misses, or FAs. For healthy participants, we used a screen with white background and dark dotted lines, while for MD participants, the background was dark and the dotted lines white. (b) Across many trials, FA responses are statistically evaluated for the degree of spatial clustering. Thresholded statistical maps show regions of visual field with a degree of clustering significantly greater than chance. Patterns of accuracy across the visual field are assessed by computing mean accuracy (hits/hits + misses) of behavioral responses within 2.5° radius of reference points, for example, in a 7 by 7 grid.

MAP samples the visual field with horizontal or vertical dotted lines, and adds “targets” to random locations of the line by creating a subtle misalignment to a subset of elements in the shape of a smooth Gaussian “bump.” Participants are instructed to locate these bumps and indicate them with a screen touch or mouse click (Fig. 1). The framework for analyzing and interpreting MAP performance is conceptually related to signal detection theory in which four possible outcomes exist: (1) Hit: a target was present and reported correctly; (2) Miss: a target was present but not selected; (3) FA: no target was present but a straight location of the line was selected as being distorted; and (4) Correct rejection: no target was presented and no response was made.

Each one of these outcomes is potentially informative of specific aspects of the participant's performance and latent visual functioning. A hit is considered as evidence for integrity of the retina and normal visual function. A miss may represent a lapse in performance (e.g., due to cognitive factors) or an inability to detect the target as a result of retinal damage or a loss in visual field sensitivity. When misses tend to accumulate in particular regions of visual space, this is strong evidence that the source of performance impairment is likely retinal because cognitive lapses should be unpredictable and distributed randomly. A FA is a distinctive event that provides evidence for metamorphopsia because the participant has reported seeing a distortion that was not actually in the stimulus array. While individuals with normal vision should make few FAs (if any) that are randomly distributed in space, individuals with metamorphopsia should tend to make many FAs that are clustered in particular regions of space that correspond to underlying retinal defects. Correct rejections, by contrast, imply that the observer saw the straight line as being straight and correctly withheld response, thereby indicating an absence of metamorphopsia. The theoretical and statistical approach of MAP analysis is therefore to evaluate the degree of spatial clustering of FAs to derive a quantitative map of pathological visual field distortions, and to evaluate the spatial distribution of hits and misses to provide a measure of visual field integrity (e.g., the accuracy of seeing and responding to salient “bump” targets).

During the MAP assessment, a set of horizontal or vertical dotted lines are drawn on the computer screen spanning ±7° across the central 14° of the visual field (Fig. 1). Line locations are sampled from a set of 18 possible horizontal or 18 possible vertical locations separated each by 0.75°. When more than one line was presented at a time, the assessment was programmed to select lines that were at least 1.5° apart so there would be no overlap between potential target bumps. Square elements composing the dotted line had a width of 0.2° and the blank distance between elements was 0.1°. A Gaussian bump (target) was randomly placed along a line by adding a small displacement to a subset of the points, which introduced a true physical distortion to the stimulus array. Gaussian bump targets were variable in size, with a width that ranged from 1.0° to 1.5° and a height of line displacement that ranged from 0.2° to 0.4°. All target sizes were chosen to be supra-threshold and easy to detect for individuals with relatively normal vision. The assessment could include a variable number of target bumps on a given trial (e.g., typically one, two, or three total targets), and the program was constrained to allow only up to one randomly positioned target on each line.

The stimulus array was displayed for 160 ms (experiment 1) or 200 ms (experiment 2) in order to minimize eye movements during display, and was followed by a blank screen that included only the fixation point as a continuous reference point. Participants were instructed to use the touch screen to quickly record locations in which a perturbation or distortion was perceived to any of the briefly flashed lines. A response was considered a hit if it was within a threshold distance (1° for healthy participants in experiment 1, 2° for patients in experiment 2) from the centroid of an actual target location; otherwise, the response was considered a FA. Participants pressed a button to indicate when all distorted regions had been manually selected to commence the following trial with an intertrial interval of 2 to 4 seconds.

We developed an adaptive sampling algorithm in which the probability of sampling a particular line location was dependent on the frequency of prior FA responses associated with that line. For example, as the test progressed, if a particular line location was presented and there was no FA on it (e.g., correct rejection), then this was taken as marginal evidence that there was no metamorphopsia anywhere along that line, and the probability of sampling that region in the future would decrease according to parameter w1. On the other hand, if a line sample did result in a FA then this was taken as evidence that there might be metamorphopsia in a region crossed by that line, and the probability of sampling that region in the future increased according to parameter w2. In simulations with a computer model (Supplementary File S1), we evaluated a range of w1 and w2 values and found a suitable balance between exploration/exploitation using w1 = −0.02 and w2 = 0.1, in which the relative influence of a FA was five times greater than a correct rejection. MAP was initiated with a uniform sampling distribution, where the probability of sampling any line was equal to 1/18 (0.055). Following each trial and the application of w1 and w2 to the sampling probability distribution, a new distribution was created by re-normalizing the sum across all possible line locations. The central idea behind the adaptive algorithm was to increase the future likelihood of sampling regions of the visual field that had already accumulated prior evidence for metamorphopsia and to hone-in on these regions more quickly.

#### Macular Integrity Assessment (MAIA) Microperimetry Exam

Microperimetry was performed (experiment 2) using the MAIA, a microperimeter with inbuilt confocal scanning laser ophthalmoscope and eye tracker.^19,20^ MAIA uses a 25-Hz eye tracker to monitor fixation, performs retinal imaging using a scanning laser ophthalmoscope, and generates targets by projecting light from a white light-emitting diode (LED) directly onto the retina. MAIA measures differential light sensitivity based on the ability of observers to reliably report seeing a brief spot of light at each sample location using a 4-2 threshold strategy in which luminance is adjusted by increments of 4 dB for increases and 2 dB for decreases. MAIA has a dynamic range of 36 dB, where a value of zero represents floor performance (poor sensitivity) and a value of 36 represents ceiling (high sensitivity). MAIA also measures fixation stability (FS) and uses an automated eye-tracking system to account for eye movements or poor fixation during stimulus presentation. FS is represented by two indices, P1 and P2, which represent the percentage of fixations within circles of 1° and 2° radius, respectively. Eyes that have a P1 value of at least 75% are classified as stable, eyes that have a P2 value of at least 75% (but are not classified as stable) are classified as relatively unstable, and eyes with P2 less than 75% are classified as unstable.

#### Amsler Grid Exam

MD patients (experiment 2) performed a paper Amsler grid exam (black lines on white background) in the laboratory with the oversight of a trained researcher (MKB). Subjective demarcations of perceptual disturbances (blurry or wavy lines or holes) on the grid (Supplementary Figure S1) were manually digitized by an experimenter (SMT) to a digital image with the same spatial resolution as the MAIA microperimetry exam associated with that eye. Specifically, each point in the digital image (e.g., a 7 by 7 image corresponding to retinal loci sampled by MAIA) was labeled as either “healthy” or “pathological” depending on whether the corresponding region (within ±1°) was demarcated by the patient in the paper version of the test.

### Procedure

#### Evaluating MAP Design Characteristics

A key feature in designing MAP was the modification and evaluation of different task characteristics to develop the most objectively effective version of the assessment. This included evaluating the impact of using different numbers of simultaneous line stimuli, different numbers of targets, and the maximum number of responses allowed per trial, as well as evaluating different methods of selecting line locations on each trial (e.g., whether locations were chosen randomly or adaptively based on prior data). We developed a realistic observer model in MATLAB (MathWorks, Natick, MA) and ran thousands of computer simulations of MAP with the model observer (Supplementary File S1). Based on results of the simulations, we found that the most efficacious design for MAP included three-line stimuli per trial and used an adaptive sampling routine for selecting line locations on each trial of the assessment. These characteristics demonstrated a significant improvement for time-efficiency of the test so that equivalent spatial accuracy in isolating regions of simulated metamorphopsia could be achieved with fewer trials.

#### Experiment 1

Experiment 1 was designed to evaluate the ability of MAP to isolate and detect simulated metamorphopsia in young healthy participants, and specifically, to evaluate a key prediction derived from the observer model simulations (Supplementary File S1). The model predicted that employing three lines versus only one line in the stimulus display would result in substantially more FA responses, and would allow MAP to converge faster (fewer trials) in measuring statistically significant clustering to indicate distortions of the visual field.

We programmed a system to simulate metamorphopsia in real-time (500 Hz) using an Eyelink 1000 tower eye-tracking device (SR Research, Mississauga, Ontario, Canada). Participants were pseudo-randomly assigned to one of four groups, where each group was assigned a ground truth region of metamorphopsia centered at one of four possible (x, y) locations including (−3, −3), (+3, +3), (−5, +5), (+5, −5) degrees relative to the fixation marker placed at (0, 0). These locations were chosen strategically to span all four quadrants of the visual field and to range in distance from fovea. During stimulus display, eye gaze location was continuously tracked binocularly and the task was performed binocularly so as to maximize eye tracking accuracy. The program waited until the participant fixated on the central point for at least 200 ms prior to displaying the stimulus array, which was shown for only 160 ms so there would be insufficient time for a saccade away from fixation. Using the continuously tracked gaze location, metamorphopsia was simulated by creating a Gaussian distortion (height = 0.5°, sigma = 0.3°) in real time to the location of any line that happened to pass through the assigned ground truth region. For example, if the participant had a ground truth of (+3, +3) degree, then if a sampled line happened to cross within 1° of this location (+3, +3) relative to actual eye gaze location, then a Gaussian bump would be added in real-time to the stimulus array in that location. On each refresh of the monitor (100 Hz), the program updated the stimulus array to include such distortions yoked to the position of eye gaze in near real-time. These Gaussian distortions yoked to eye position were similar in appearance to actual target bumps, but for the purposes of analysis, responses to the yoked targets were registered as FAs.

Participants performed the task under two conditions presented in pseudorandom order across participants. Condition 1 presented only one line per trial with one randomly placed target every trial (analogous to the PHP test). Condition 2 presented three lines per trial with up to three randomly placed targets (max of one target per each line), with a probability of 0.7 for one target present, 0.2 for two targets, and 0.1 for three targets. Participants were instructed to “press the screen in all locations where you perceive a distortion to any line.” The trial was completed either when participants pressed a button on the screen to indicate that all perceived distortions had been selected, or when the maximum number of responses allowed was reached (up to four per trial). Participants completed 64 total trials for each condition, which took up to 10 minutes to complete. A training procedure prior to the experiment helped to familiarize participants to the task and ensured they could respond with at least 90% accuracy to target bumps.

#### Experiment 2

Experiment 2 was designed to evaluate repeatability of MAP performance in patients diagnosed with macular disease, and to statistically compare visual field maps derived from MAP with those derived from MAIA microperimetry and the Amsler grid. Data were initially collected from three patients (MD1–MD3) who performed the MAP assessment twice (separated by 12 weeks) and who also had records of a microperimetry exam performed within the last 4 years. Amsler grid data were not available for these three patients. We subsequently collected data from five additional patients in which the protocol was modified to acquire more proximal MAIA microperimetry and Amsler grid exams obtained during the same test and retest sessions as MAP. In both cohorts, there was approximately a 12-week period between visual assessments.

The MAP procedure was similar to experiment 1 but with a few modifications. We employed a single MAP design that had three lines per trial, adaptive sampling, and a total of 64 trials. Given the older age of MD patients and general level of visual impairment, the stimulus was presented for 200 ms (versus 160 ms used in experiment 1). Lastly, a trained experimenter remained in the room with the participant during testing. The participant was asked to touch the location(s) on the screen where s/he saw a distortion in the sampled lines, and then a researcher would operate the mouse to click on those points. Some of the patients had difficulty in navigating the mouse cursor, and a touch screen monitor was not available at the testing site, so this approach seemed a reasonable compromise. During testing, the participant wore an eye patch over the eye that was not being tested.

For three patients, the MAIA microperimetry exam was conducted several months prior to the recruitment of the participants. Specifically, for patients MD1, MD2, and MD3 microperimetry was conducted 13, 33, and 44 months prior to the current experiment, respectively. These examinations used MAIA's Goldmann-style 10°/37-point meridional test pattern centered on the fovea to assess full thresholds at 37 retinal loci in a radial pattern. For patients MD4 to MD8, the MAIA exam was conducted on the same days as MAP and used the 61-point linear test pattern that included 49 loci within the central 14° of the visual field corresponding to an equidistant 7 by 7 square grid spanning from −6 to 6° in steps of 2°. All the tests were conducted monocularly in a quiet, darkened room. Before testing, participants underwent a brief period of training, allowing them to familiarize themselves with the target stimulus and practice correct operation of the response button. In total, testing took approximately 40 minutes to 1 hour, including a short (3–5 minutes) break between each successive test.

## Analysis

### Analyzing MAP Responses

#### Computing MAP Visual Field Accuracy Maps

We evaluated the distribution of hits and misses in the MAP assessment to derive spatial maps of visual field integrity based on task accuracy. We used the MAIA sampling scheme associated with each particular eye (37-point radial scheme for MD1–MD3, or 49-point linear scheme for MD4–MD8) as a reference for mapping MAP accuracy to a sparse grid that could be directly compared to MAIA measurements (Fig. 1b). For each retinal locus in the MAIA grid, we found all the hits and misses within a 2° radius and computed accuracy as *hits*/(*hits* + *misses*). The MAP accuracy value at each locus was only considered valid if at least four targets (e.g., total of hits and misses) had been presented within the 2° radius circular region centered on that locus; otherwise this value in the MAP was represented as NaN. Due to random sampling, there were a few loci with insufficient (e.g., less than four) target samples (6.3% of all possible loci) and these loci were disregarded in subsequent analyses.

#### Computing MAP Visual Field Distortion Maps

MAP FA responses were analyzed with a bootstrapped clustering technique to identify retinal regions suspected of metamorphopsia. Smoothed statistical maps of visual field distortions were computed by examining the probability associated with the spatial clustering of FAs (Fig. 1b). The procedure for computing the distortion map was as follows: (1) create an image of zeros (160 by 160) spanning + 8° of the visual field from center, where each pixel represents 1/10°, (2) replace a value of 1 for each of *n* FA locations in the map, (3) smooth the image by convolving with a 2d Gaussian filter (sigma = 10 pix, equivalent to 1°), and (4) threshold the map according to an appropriate statistical criterion. Any region that exceeded the criterion, by definition, had clustering of FAs that was unlikely to have occurred by chance and therefore indicative of pathological retinal distortion.

To establish an appropriate criterion for evaluating statistical significance, we used a random permutation test to assess the distribution of maximum values expected by chance (e.g., the method of maximums) using an identical analysis pipeline on an equivalent number of locations that were instead chosen uniformly and randomly across visual space. For example, we would simulate a thousand examples comprising *n* random point locations and store the maximum value from the resulting smoothed map to create a null distribution of maximums. Intuitively, maximum values of the simulated map tend to be high only when there is clustering of points by chance; otherwise, the maximum values will tend to be quite low. We used the 95th percentile of this null distribution as the threshold for evaluating statistical significance.

The thresholded FA distortion maps therefore provide an estimate of the location and extent of regions suspected of metamorphopsia. In experiment 2, in order to facilitate direct comparisons with MAIA and Amsler grid outcome measures (analogous to the computation of MAP accuracy maps at particular loci) we resampled the distortion maps to the same resolution as the MAIA reference grid. This was performed in a fashion similar to the Amsler grid data, where each point in the reference grid (e.g., the loci sampled by MAIA) was labeled as either “healthy” or “pathological” depending on whether the corresponding region (within ±1°) reached statistical significance in the MAP FA distortion map.

### Discriminating Eyes With Severe Pathology

A primary measure of the clinical utility of a visual assessment designed for MD is its ability to distinguish eyes with minor pathology from eyes presenting severe pathology. We used MAIA threshold measurements as a relevant clinical standard for classifying the severity of visual pathology. Severe pathology was defined as having at least three MAIA threshold measurements (out of either 37 or 49 loci) that were each less than 5 dB, which would be associated with a regional loss of visual sensitivity and strongly indicative of a scotoma or severe retinal damage. We used receiver operator characteristic (ROC) analysis to compute the sensitivity and specificity of MAP accuracy values in discriminating eyes with and without severe pathology.

We also evaluated the extent to which MAP FA distortion maps could discriminate between healthy versus pathological eyes defined, instead, by the presence of a positive Amsler grid test (e.g., whether or not the participant subjectively demarcated any region at all on the Amsler grid). We report the accuracy and sensitivity of the classification of Amsler grid pathology using the binary variable specifying whether or not there was any significant FA clustering in the MAP distortion map.

### Test–Retest Reliability of Visual Field Patterns

We evaluated test–retest reliability of MAP, MAIA, and Amsler grid visual field patterns using two complementary techniques. We evaluated Pearson product–moment correlation coefficients and intraclass correlations (ICCs) to quantify the linear relationship between the first and second tests. While the correlation coefficients reveal the strength of positive relationship between two measurements, they do not quantify the absolute level of agreement. We therefore performed parametric Bland-Altman analysis on test–retest measures for MAIA thresholds and for MAP accuracy values to examine the bias (mean of test–retest differences), upper and lower 95% limits of agreement (ULA, LLA), and coefficient of reproducibility (COR) defined as 1.96 times the standard deviation of test–retest differences. The FA distortion maps and Amsler grid assessments provide a binary outcome measure for loci in the visual field (healthy “undistorted” versus “distorted”), which is incompatible with the Bland-Altman method. Direct comparison of measurements between the different assessment types, which are measured on inherently different spatial scales, was achieved by resampling the Amsler grid and MAP results to the same retinal loci sampled by MAIA microperimetry. Test–retest reliability analysis was therefore targeted at establishing the repeatability of spatial visual field patterns across these specific loci.

### Interassessment Consistency of Visual Field Patterns

We evaluated the consistency of measurements between pairs of different assessments (e.g., between MAP and MAIA or between MAP and Amsler) using Pearson correlation coefficients and ICC of measurements across all retinal loci. We did not measure strict agreement between the assessments because the measurement units were on very different scales (proportion correct for MAP, threshold decibels for MAIA, binary labels for Amsler); however, we expected the general consistency between assessments to be suitably captured by evaluating the strength of linear relationships between the outcome variables. We evaluated the consistency between MAP accuracy values and MAIA microperimetry thresholds, expecting to find a positive linear relationship due to the fact that low microperimetry thresholds are strongly associated with anatomical defects (e.g., scotoma), and observers should therefore have an impaired ability to detect MAP targets presented to regions affected by scotoma.

Similarly, we examined the consistency between MAP FA distortion maps and Amsler grid maps in terms linear correlation coefficients and ICC. We evaluated consistency between the exams at two levels including (1) the binary labels (healthy versus pathological) from each exam associated with individual visual field loci, and (2) the total regional area demarcated on the Amsler grid and the area found to be statistically significant in the MAP FA distortion maps.

## Results

### Experiment 1

Experiment 1 was designed to compare performance in the one-line versus three-line conditions with respect to (1) how often participants made responses to artificially distorted regions yoked to eye gaze position (designated as FAs for purposes of analysis), (2) how accurate the resulting maps were in localizing the simulated metamorphopsia region, and (3) overall how many individuals across the sample provided a positive result in detecting the presence of metamorphopsia within the 64-trial limit. A positive test was determined by whether there was a statistically significant spatial clustering in the map. The estimate of metamorphopsia location was determined by the location of the peak in the statistical map, and spatial accuracy was calculated as the Euclidean distance between the centroid of the simulated distortion (the “ground truth”) and the peak location. FA rate was computed as the total number of FAs divided by the number of trials.

**Figure 2:**
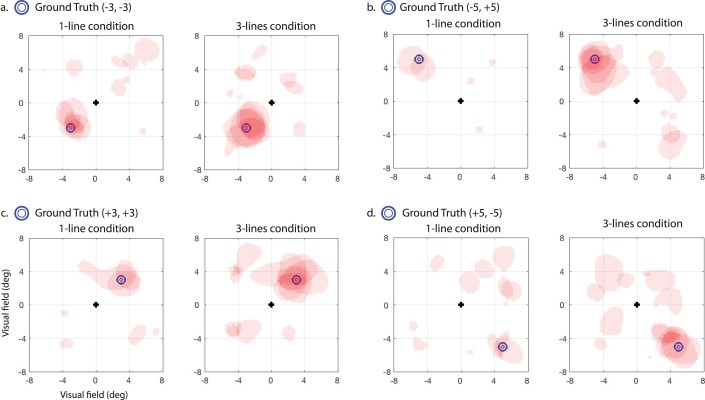
Results for simulated metamorphopsia with healthy participants in experiment 1, with thresholded statistical maps of FA clustering overlaid from each participant, and with ground truth shown for reference (blue circle markers), which represents the assigned ground truth location for four different subject cohorts (a through d). The left panel in a to d represents maps from the one-line per trial condition, and the right panel represents three-lines per trial condition. The reddish pink regions are statistically significant (P < 0.05) as assessed with permutation testing, and have transparency so that darker red regions indicate more overlap across participants.

Thresholded statistical maps for each participant are shown overlaid together with ground truth (blue circle markers) for visual comparison in Figure 2. Since our procedure simulated metamorphopsia in the location centered on the blue markers, as expected, we observed that a majority of significant FA clusters (reddish pink regions) were found surrounding or adjacent to the blue markers. As a group, 90% of the participants in the three-line condition reached the criterion for a statistically significant spatial clustering within the 64-trial limit, whereas in the one-line condition this was the case for only 45% of the participants. This result has a strong similarity to results of the simulated observer model (Supplementary File S1), which showed that it took only 50 trials on average to reach criterion (defined as a positive result with significant clustering) for the three-lines condition, but took over 100 trials on average to reach criterion in the one-line condition.

The higher likelihood of detecting simulated metamorphopsia was driven by the fact that there were significantly more FAs measured in the three-line condition (mean = 0.59 FAs/trial, SD = 0.36) in comparison to the one-line condition (mean = 0.14 FAs/trial, SD = 0.18) (*t*_38_ = 4.98, *P* = 0.013). There was, however, no significant difference in spatial accuracy for cases in which metamorphopsia was successfully detected between the three-line (mean = 1.0°, SD = 0.46) and one-line (mean = 0.80, SD = 0.52) conditions (*t*_25_ = .49, *P* = 0.65). This result also mirrors the outcome of observer model simulations (Supplementary File S1), showing that localization accuracy was comparable between the two conditions. Thus, the ability of the three-line condition to better detect metamorphopsia was driven principally by the fact that it produced a higher rate of FAs.

These results show that the theoretical gains in time-efficiency predicted by the ideal observer model were largely confirmed in the results of experiment 1 with simulated metamorphopsia in young healthy participants. The results also demonstrate, in principle, that the design and analysis technique employed by MAP has the potential to measure real pathological distortions in MD patients through quantitative maps of FA clustering.

### Experiment 2

Experiment 2 was designed to assess the suitability of MAP in measuring functional visual field abilities in observers diagnosed with various types of macular disease (Table 1) and with heterogeneous patterns of retinal damage. Outcome measures for each patient, eye, and test number are reported in Table 2 for MAIA, MAP, and Amsler grid.

**Table 2:**
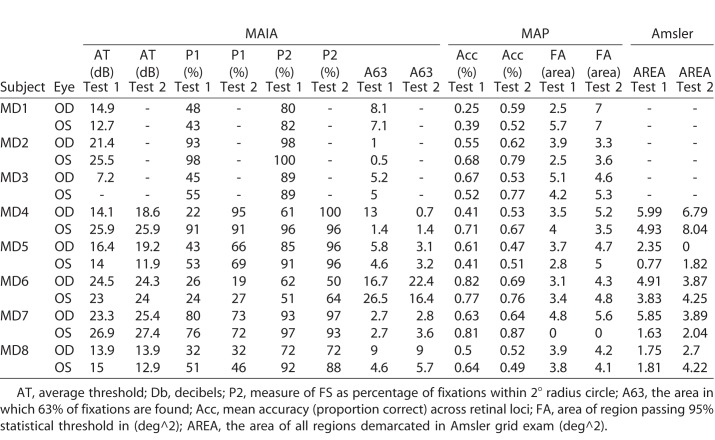
Measurements Obtained by MAIA Microperimetry, MAP, and Amsler Grid for Each Participant, Each Eye Examined, and Each of Two Tests Taken 12 Weeks Apart

**Figure 3:**
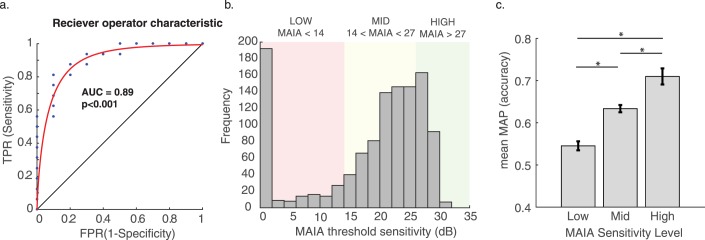
(a) ROC curve for classifying eyes labeled as pathological versus severely pathological (defined from MAIA microperimetry thresholds) using mean MAP accuracy measurements. (b) Distribution of MAIA sensitivity values across all retinal loci from all eyes in the study, where three discrete bins are defined to represent low, mid, and high level sensitivity. (c) Mean MAP accuracy and standard error across loci categorized as low, mid, and high based on MAIA sensitivity level. *P < 0.0001.

We performed ROC analysis to distinguish eyes characterized as having severe visual pathology (Fig. 3a), defined as having at least three MAIA threshold measurements less than 5 dB, which would be highly indicative of a scotoma or regional loss of visual sensitivity. ROC discriminability (*n* = 26) was statistically significant (area under the curve = 0.89 ±0.06, *P* < 0.0001), and results showed a sensitivity of 0.81 and specificity of 0.90. The best cutoff value for mean MAP accuracy was 0.61, indicating that a mean accuracy less than 61% was highly diagnostic of the presence/absence of severe visual pathology defined on the basis of MAIA threshold sensitivity values.

We extended this analysis by assessing the relationship between MAP accuracy measurements and MAIA sensitivity thresholds categorized into three discrete bins (Fig. 3c), across all retinal loci. A subset of loci across all eyes (*n* = 127) showed relatively high function and mean MAIA values greater than 27 dB, another subset (*n* = 753) showed intermediate sensitivity greater than 14 dB (but less than 27 dB), and another subset (*n* = 532) showed low mean sensitivity less than 14 dB (Fig. 3b). The mean MAP accuracy value for these three MAIA levels was 0.54, 0.63, and 0.71, respectively, which were all significantly different from each other (*t*-tests, all *P*s < 0.0001). This suggests a strong statistical relationship between MAP accuracy and MAIA thresholds, not just at the level of individual eyes (as revealed by ROC analysis), but also at the finer scale of individual retinal loci.

Next we examined the ability of MAP FA distortion maps to distinguish eyes showing a positive Amsler grid exam. Nineteen out of 20 eye exams showed a positive Amsler grid and 18 out of 20 MAP assessments showed significant spatial clustering of FAs to indicate metamorphopsia. Overall the mean accuracy of classification of eyes as having positive Amsler grid assessment was 0.85 with a sensitivity of 0.89. In general, this was a group of subjects selected on the basis of having macular disease (19/20 Amsler exams showed visual disturbance), and MAP FA distortion maps were successful in confirming this diagnosis with a positive test for majority of eyes tested (18/20).

Test–retest analysis (Table 3) showed that all four types of visual field measurement had statistically significant repeatability according to linear correlation analyses. MAIA microperimetry showed the strongest test–retest reliability (*r*(488) = 0.65, *P* < 0.0001), which is perhaps not surprising given that this is a clinical-grade assessment that uses a scanning laser ophthalmoscope and eye tracker in real time to help ensure that stimuli are presented to precise locations on the retina. The Amsler grid also produced reliable binary measurements (*r*(488) = 0.30, *P* < 0.001) of whether individual loci were classified as healthy or pathological. From MAP, both the thresholded FA distortion maps and the accuracy maps showed a similar level of reliability (*r*(704) = 0.38, *P* < 0.0001 and *r*(628) = 0.42, *P* < 0.0001, respectively). In terms of absolute agreement of measurements across individual loci, the Bland-Altman analysis revealed moderate agreement with a rather high COR for both MAIA sensitivity measurements (bias = 2.08 dB, ULA = 17.75 dB, LLA = −13.58 dB, COR = 15.67) and for MAP accuracy (bias = 0.04, ULA = 0.55, LLA = −0.47, COR = 0.51).

**Table 3:**
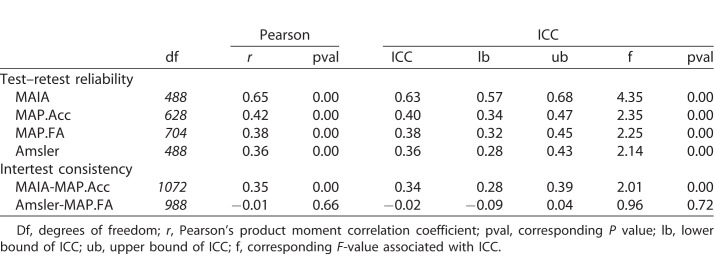
Pearson Correlation Coefficients and ICCs Representing Test–Retest Reliability and Interassessment Consistency of Visual Field Measurements for MAIA Threshold Sensitivity, MAP Accuracy (MAP.Acc), MAP FA Distortions (MAP.FA), and Subjective Amsler Grid Demarcations

Next we examined the linear relationship between MAIA sensitivity measurements and MAP accuracy measurements across visual field loci to evaluate whether these two distinct exams provided similar measurements. MAIA measures light sensitivity and MAP measures the ability to accurately detect the location of target bumps, but both tasks depend on proper visual sensitivity and functioning to achieve high values. We found a strong and statistically significant relationship between these two measures (*r*(1072) = 0.35, *P* < 0.0001), demonstrating that the spatial patterning of MAIA sensitivity values was broadly consistent with accuracy measurements obtained by MAP. To account for the fact that eye label (left, right) and exam number (test 1, test 2) were likely correlated across measurements due to the fact that these variables were nested within subjects, we followed up the linear correlation analysis (which is a conservative test that assumes independence of measurements) by implementing a multilevel mixed-effects linear model, with eye and exam number nested within subjects as random effect variables, and MAP accuracy values as a fixed effect variable to explain MAIA sensitivity. Even after accounting for eye and exam number nested within subjects, MAP accuracy values remained a highly significant predictor of MAIA values, *t*(1071.9) = 5.03, *P* < 0.00001. The full nested mixed-effects model had an adjusted *r*^2^ of 0.38, whereas a simple fixed-effects linear regression model had an adjusted *r*^2^ of 0.12.

By contrast, we did not find a statistically significant relationship between Amsler grid results and MAP FA distortion maps in terms of visual field map patterns. This is interesting because while the Amsler grid test and MAP FA maps each showed strong repeatability from test to retest within themselves, there was no statistical relationship between their measurements (*r*(988) = −0.01, *P* = 0.66) at the level of individual retinal loci. We also examined whether there was a linear relationship between the two exams in terms of the total area of the visual field demarcated as pathological (i.e., regions demarcated subjectively via Amsler versus demarcated via statistical FA clustering analysis for MAP; see Table 2). We found no statistically significant relationship between the estimated extent of pathology derived from Amsler and MAP, *r*(18) = 0.33, *P* = 0.15. Therefore, while each assessment is designed theoretically to estimate similar underlying constructs (i.e., pathological distortions), the two exams appear to be measuring distinct, but internally repeatable aspects of visual function.

### Selected Case Studies

Next we report details of specific case studies of selected patients enrolled in the study. For patients MD1 to MD4 and MD8, we report individual case studies in Supplementary File S2. In the following section, we selected three patients as representative examples to discuss correspondence between anatomical fundus images and outcomes measures from MAP and MAIA microperimetry.

#### Patient MD5, Stargardt's

**Figure 4:**
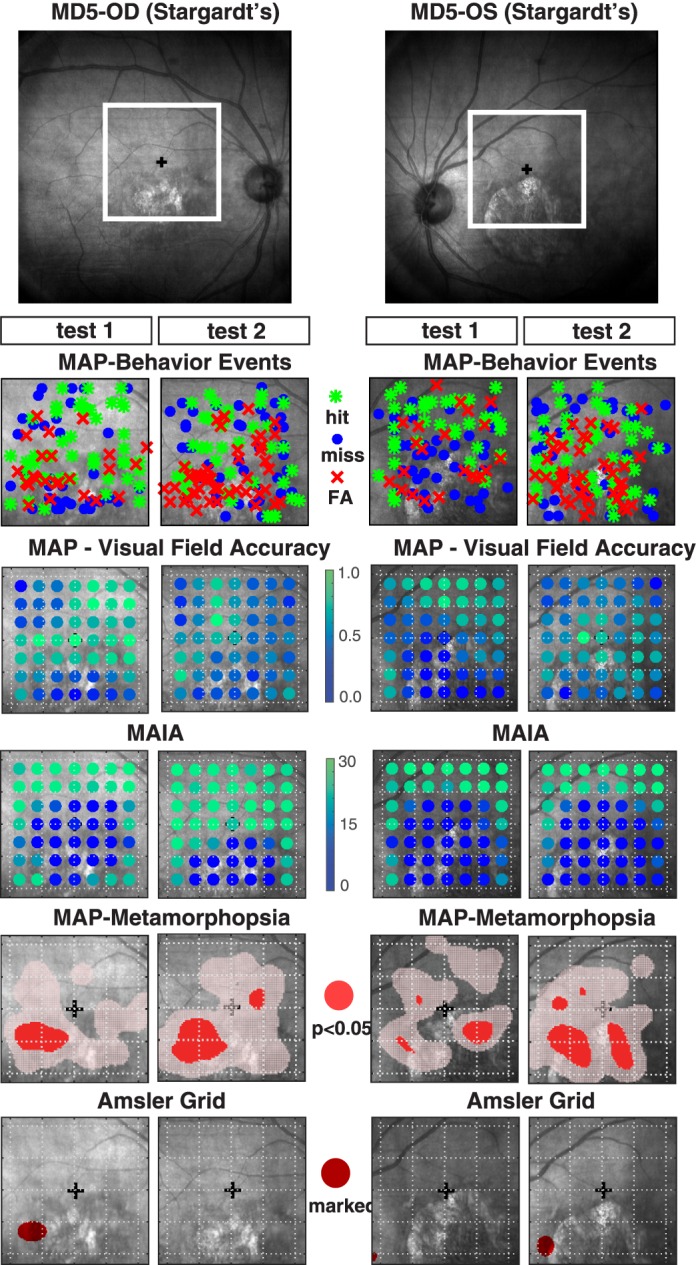
(Upper panel) Fundus image of OD and OS for patient MD5 diagnosed with Stargardt's disease with box identifying the central 14° of the visual field centered on the fovea. (Lower panels) Test measurements from test 1 and test 2 associated with MAP and MAIA, overlaid on the corresponding location of the retina centered on the fovea. MAP data were scaled, inverted, and then mirror reversed to transform from Cartesian coordinates to approximate retinal fundus image coordinates. The first lower panel shows MAP behavioral responses including FAs (red dots), hits (green dots), and misses (blue dots); the second lower panel shows MAP accuracy values resampled to the same resolution as the reference MAIA grid for direct comparison of measurements across retinal loci between the two tests. The third lower panel shows MAIA threshold sensitivity values. The fourth lower panel shows thresholded FA distortion maps, where regions marked red indicate a statistically significant degree of clustering for FA responses. The fifth lower panel shows a mirror-reversed projection of subjective Amsler grid demarcations onto the fundus image for easier visual comparison the FA distortion maps.

Patient MD5 is a 62-year-old diagnosed with bilateral Stargardt's disease in which a substantial deformation of the retina is visible in the fundus image of OD and OS, with a scotoma encompassing nearly the entire inferior region of the macula, corresponding to the upper region of the visual field (the central 14° is outlined with a white box in Fig. 4). MAIA microperimetry confirms this observation showing a near complete loss of visual sensitivity in the inferior macula of both eyes, a result that was highly repeatable from test 1 to test 2. MAP behavior responses showed a consistent pattern with MAIA, showing a tendency for more misses and FAs occurring in the upper visual field (inferior macula region). The MAP accuracy maps showed relatively poor accuracy across the visual field, with a slight tendency for lower accuracy in corresponding regions of the inferior macula, but the boundary is not as well defined as with MAIA. MAP metamorphopsia distortion maps detected significant clustering in lateral regions of the inferior macula, right along the boundary and partially overlapping the scotoma region. The thresholded distortion maps were quite repeatable from test 1 to test 2, with OD showing a distinct region centered 3° temporal and 3° inferior relative to the fovea, with a radius of approximately 2°. This result is very consistent with the region demarcated on the test 1 of the Amsler grid; however, on test 2 the patient failed to demarcate any region of the Amsler grid. Due to consistent reporting of FAs in this region throughout both examinations, there is strong likelihood of visual disturbance (e.g., metamorphopsia) in this region on the boundary of the scotoma. OS also showed a repeatable pattern of metamorphopsia on the temporal and nasal boundaries of the anatomical scotoma in the inferior macula. However, this result was inconsistent with subjective demarcations on the OS Amsler grid for tests 1 and 2, in which the patient indicated only very small distortions at the nasal boundary (6°–7°) of the inferior macula. In summary, MAP accuracy and FA distortion maps showed quite strong repeatability for both eyes of MD5, and a strong correspondence with retinal fundus images and patterns of MAIA sensitivity thresholds. However, there was only low-to-moderate consistency between FA distortion maps and subjective Amsler grid reports.

#### Patient MD6, Myopic Degeneration

**Figure 5:**
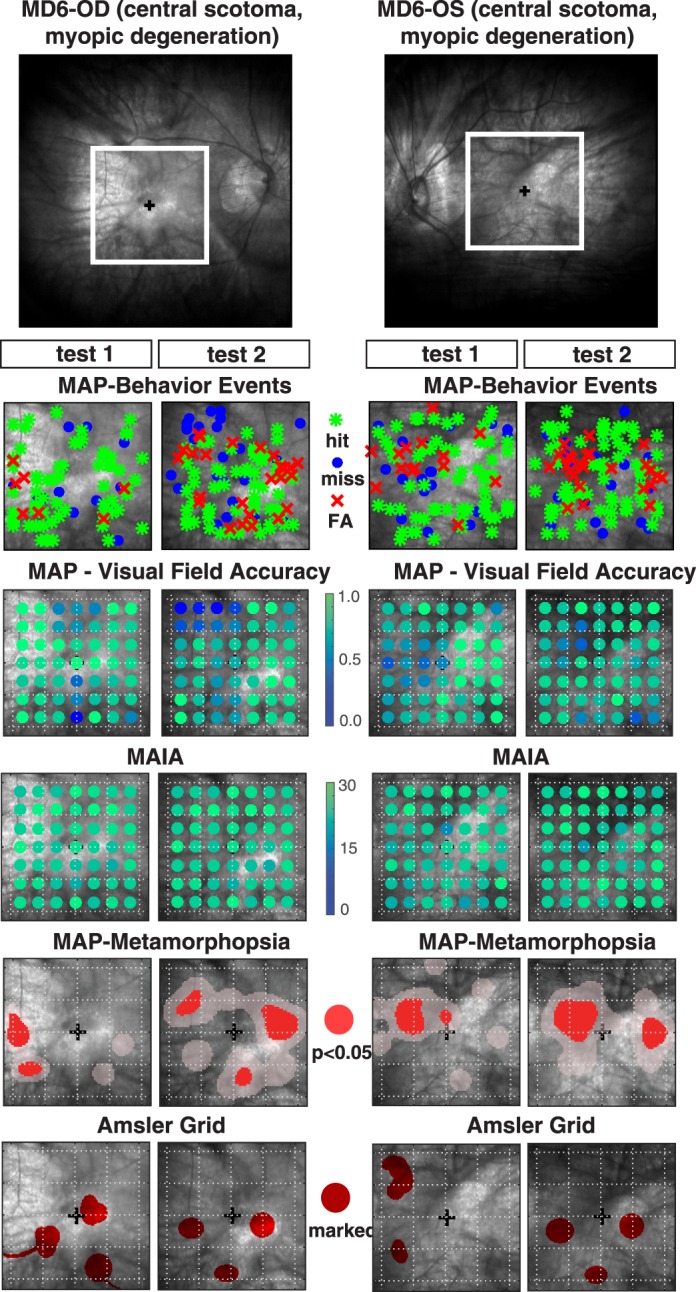
(Upper panel) Fundus image of OD and OS for patient MD6 diagnosed with myopic degeneration and central scotoma. The lower panels are the same as described in Figure 4.

Patient MD6 is a 69-year-old diagnosed with bilateral myopic degeneration in which the central scotoma is not well-defined in the fundus images (Fig. 5). MAIA microperimetry showed a relatively good level of visual sensitivity across the entire visual field, with no single locus showing a complete loss in visual sensitivity (e.g., all MAIA thresholds > 15 dB) and an average threshold of 24 ± 2.4 dB across both eyes on tests 1 and 2. MAP behavior responses showed a similar pattern in which there were few misses that did not tend to cluster in visual space, and MAP accuracy was relatively high across the entire visual field, with an average accuracy of 0.76 ± 0.18 across both eyes on tests 1 and 2. Both MAP and MAIA indicate moderate impairment of visual sensitivity and relatively intact visual field integrity. The thresholded distortion maps did show distinct patterns of pathological distortion that were very repeatable from test 1 to test 2 of each eye. In particular, for eye OS there was significant clustering of FAs between 2° and 4° nasal and 0° to 3° of the superior macula (corresponding to lower-right of visual field), and a radius of about 1° to 2°. There was another area of significant clustering in the middle temporal macula revealed in test 2. The Amsler grid showed a similar pattern on test 1 and test 2 that was quite consistent with FA distortion maps, with multiple distributed foci marked as subjective distortions. For eye OD, there was less consistency in the location of thresholded regions from test 1 to test 2, but across both exams there was a tendency for FAs to occur in a circular pattern forming a ring of metamorphopsia with radius of 3° to 6° centered at the fovea. This spatial pattern with multiple distributed foci was also demonstrated quite strikingly in the Amsler grid reports for tests 1 and 2, but was found to be shifted slightly inferior relative to the FA distortion maps. In summary, MAP accuracy and MAIA sensitivity thresholds showed a similar pattern of intact visual function across the entire visual field, yet MAP FA distortion maps revealed repeatable patterns to indicate metamorphopsia in parafoveal regions that were roughly consistent with the distributed pattern of multiple Amsler grid demarcations of subjective distortion.

#### Patient MD7, Dry Age-Related MD (AMD)

**Figure 6:**
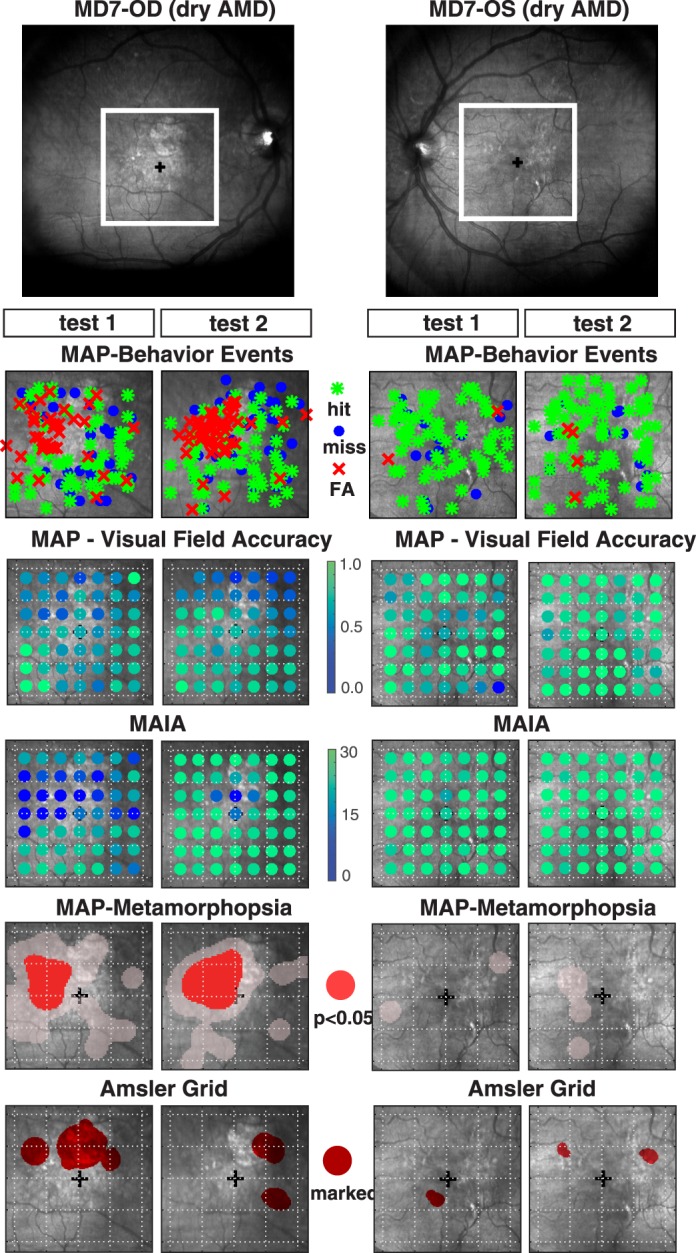
(Upper panel) Fundus image of OD and OS for patient MD7 diagnosed with dry AMD. The lower panels are the same as described in Figure 4.

Patient MD7 is a 62-year-old diagnosed with dry AMD and good visual acuity (20/25). MAIA thresholds for eye OD showed a substantial loss of sensitivity in the superior-temporal part of the macula (the lower right of the visual field), suggesting a visual defect (i.e., scotoma) in this specific region (Fig. 6). By contrast, MAIA thresholds for eye OS were high across the entire visual field on tests 1 and 2. MAP accuracy maps also showed a general reduction of accuracy in the superior macula for OD and correspondingly broad levels of high accuracy for OS on tests 1 and 2. This outcome highlights the tight relationship between MAIA microperimetry thresholds and MAP accuracy measurements at both the level of mean performance for individual eyes, and at a finer scale including spatial patterns across retinal loci. Interestingly, for OD the FA distortion maps showed a highly repeatable pattern of significant FA clustering in and around the boundary of the scotoma region. Likewise, the Amsler grid showed large demarcations of subjective distortion in the superior macula in and around the location of scotoma. The degree of clustering of FA responses in this region, together with the reduction of MAP accuracy, strongly suggests severe pathology in this area of the macula. By contrast, eye OS generated very few FAs during the MAP exam and had high visual sensitivity, indicating an integral OS visual field. While the FA distortion maps revealed no significant degree of clustering for OS, the Amsler grid did show a few very small areas demarcated as subjectively distorted.

## Discussion

MD is a progressive disease affecting central vision and represents the primary cause of blindness in western countries. Early diagnosis and constant monitoring may be crucial for both prompt intervention and actionable assessment of progressive disease states. Indeed, recent studies have shown that timely interventions can be critically important in these patients,^21^ with new drugs available that can prevent further vision loss^21–24^ and improve vision in approximately one-third of MD patients. The Amsler grid is commonly used as a simple and practical way to screen for MD and to track MD symptoms via self-administration at home. However, the Amsler grid has limitations that make it unsuited for quantitatively measuring and tracking changes in visual functioning. In this paper, we propose a psychophysical test called MAP that is designed to localize and quantify central visual field deficits (e.g., metamorphopsia and scotoma) that are associated with various types of retinal disease including MD.^25–27^

We assessed the theoretical ability of MAP to quantitatively map simulated visual field disturbances by evaluating its accuracy and efficiency using simulations with a computer model observer. In experiment 1, we empirically tested predictions from this model, and confirmed that the most efficacious design for MAP included three-line stimuli per trial and adaptive line sampling. With respect to procedures that use only one line stimulus per trial (e.g., the PHP test), MAP with three lines significantly increased testing efficiency by increasing the rate of FAs, and reduced the number of trials needed to isolate regions suspected of metamorphopsia. A principal feature and benefit of assessing MAP with the observer model and the healthy vision population in experiment 1 was to be able to control and specify a priori the location and extent (the ground truth) of the distorted region. For example, there is inherent difficulty in determining the *true* retinal regions associated with metamorphopsia in patients due to the subjective, perceptual nature of the disturbance, as well as idiosyncratic manifestations of the disease in each individual as well as changes over time.

It is important to note that despite surface similarity in stimulus appearance (e.g., dotted lines with target bump displacements) between PHP^16^ and MAP, there are several significant distinguishing factors. The PHP allows exactly one response per trial with the understanding that true stimulus distortions will compete with perceptual distortions in other locations, and the observer will likely choose the most severe distortion (hence the “preferential” aspect of the test). By comparison, on any given trial, an observer in MAP responds to all perceived distortions, including true targets and pathological distortions. The analytical basis of MAP is instead to examine the spatial patterning and statistical clustering of behavioral responses (hits, misses, and FAs) as a means of estimating spatial maps of visual field integrity and visual field distortion. Intuitively, MAP would benefit from employing more line stimuli in order to maximize the number of opportunities for these types of behavioral responses to occur, thereby increasing the amount of information gained on every trial. A quick procedure would be of significant utility since the patient might be able to quickly check their vision on a daily basis.

In experiment 2, we tested MAP in eight MD patients (16 different eyes). Results showed good test–retest reliability, as well as consistency between MAP measurements and MAIA sensitivity thresholds. In fact, mean MAP accuracy measurements were able to distinguish severe visual pathology, determined independently from MAIA microperimetry values, with high accuracy, sensitivity and specificity equal to 0.89, 0.81, and 0.90, respectively. MAIA is a high precision technology that would obviously not be replaced by a psychophysical examination like MAP; however, the ability of MAP to approximate retinal patterns of MAIA sensitivity measurements suggests that MAP could serve as a useful surrogate method for obtaining frequent at-home measurements of visual field integrity between visits to the clinic. While promising, these preliminary data should be interpreted with caution, as more research in nonclinical groups will be necessary to build normative data sets and to demonstrate the ability of MAP on a larger scale to discriminate the degree of visual pathology with both high sensitivity and specificity for low-vision patients and healthy vision individuals.

A promising feature of MAP is the potential ability to provide quantitative estimates of the location and extent of retinal areas affected by metamorphopsia. For several MD patients, our work illustrates strong test–retest reliability of visual field distortion maps estimated from clustering of FA responses accumulated during the MAP assessment. Further, the anatomical estimates of metamorphopsia in these participants were generally found within or along the borders of the scotoma, which is consistent with the literature on metamorphopsia.^26,28^ Late-stage MD patients with central visual field loss often rely more upon their peripheral vision and develop a preferred retinal locus (PRL), an eccentric fixation point used in substitution of the fovea.^29^ However, even for late stage MD, for which the treatment consists of improving vision in the periphery, and learning to use a sensitive or favorably located peripheral fixation spot (trained retinal locus [TRL]^30^), metamorphopsia mapping may have beneficial use. TRL localization is often based on anatomical examination of the fundus of the eye and/or sensitivity maps,^31^ which may fail to reveal metamorphopsias that could hinder the chosen TRL.

Interestingly, although the FA distortion maps were internally reliable in terms of test–retest, there was a lack of strong evidence for spatial consistency between MAP estimates of metamorphopsia and those obtained subjectively from Amsler grid examinations. The idea behind the Amsler grid is to track retinal defects via patterning of manual demarcations of subjectively perceived visual distortions. However, we found insignificant relationship to patterns of distortions indicated during the MAP exam. This discrepancy may be due to methodological differences including the fact that the Amsler grid is shown continuously on the entire visual field and allows patients to move their eyes around as they draw/circle the areas of visual distortion, whereas MAP isolates visual pathologies from statistical clustering of FAs to line stimuli presented so briefly as to prohibit eye movements away from fixation. Since it is difficult to establish a ground truth for distorted vision and subtle retinal deformations in MD patients, it remains unclear whether MAP provides relatively better diagnostic or clinically useful information about metamorphopsia.

Finally, a possible additional element that might have contributed to the observed discrepancy is the specific version of the Amsler grid used in this study, the modified Amsler grid, where a black grid is presented over a white background (differently from the original Amsler grid, where a white grid is presented over a black background). While the modified version has practical advantages (i.e., it makes it easier to mark with a pencil the regions of distortion), it has also been shown to be less sensitive with respect to its original version.^10^ Additional data should be collected in future studies with patient monitoring longitudinally across several weeks or months in order to address this question more fully.

Taken together, these results demonstrate that MAP presents a number of potential advantages and features with respect to current techniques. Presenting multiple lines per trial with adaptive sampling reduced the effective duration of the assessment, and our analysis of response clustering allowed simultaneous parsing of the visual field into three functionally relevant categories. The spatial resolution of MAP could allow combining MAP with microperimetry to project measurements of visual field functional integrity onto the anatomical surface of the retina to get a more composite view of visual abilities beyond light sensitivity.

The current limitations of MAP are the result of common difficulties in dealing with clinical populations in general, and with MD patients in particular. A typical characteristic of MD, the loss of central vision, might lead to poor fixation stability in the PRL, thus limiting the spatial accuracy of the visual field mapping. To help overcome this issue in principal, we integrated a gaze contingent display in experiment 1 with healthy observers to allow presentation of the stimuli in specific locations relative to fixation. In future work, gaze contingency of stimulus display could also be incorporated with patients to have better control of retinal stimulation during MAP assessment. Another known difficulty is due to the older age of AMD patients in general, which could induce low compliance and a lower tolerance to fatigue. We therefore plan to evaluate different adaptive procedures tailored to various patient populations to hone-in on regions suspected of scotoma and/or metamorphopsia as quickly and efficiently as possible. Future directions would also involve adapting MAP into accessible programs that can be run on a laptop or tablet computer, allowing for a greater number of participants and the possibility of testing rehabilitation protocols for patients performing the assessment at home. Moreover, the MAP test could benefit in the future from clinicians' and ophthalmologists' feedback on implementation with various heterogeneous groups of patients.

In summary, MAP shows the potential to be an effective and quick tool for measuring functional visual abilities and capturing the presence and location of visual field disturbances. Indeed, a timely diagnosis can be crucial in MD intervention, since new treatments for MD are available that can preserve residual vision in these patients.^32^ Further research is needed to examine whether MAP may be useful as a diagnostic tool for detecting early MD and whether MAP measurements may be useful for tracking disease progression over time to detect the onset of CNV, or wet AMD, which can cause sudden and pervasive damage to the retina. Though more research will be needed in a larger clinical sample, as well as with healthy individuals and those at risk for MD, the present results demonstrate the potential of MAP as a diagnostic and rehabilitative tool to monitor the status of visual retinal functions, which we expect may help reduce costs and increase accessibility by comparison to other exams that require more expensive and dedicated equipment.

## Supplementary Material

Supplement 1

Supplement 2

Supplement 3
