# Supplementary material for: Multi-line Adaptive Perimetry (MAP): A New Procedure for Quantifying Visual Field Integrity for Rapid Assessment of Macular Diseases
Source: Transl Vis Sci Technol. 2018 Oct 16;7(5):28. doi: 10.1167/tvst.7.5.28 (PMC6192464; doi:10.1167/tvst.7.5.28)
Supplement: Supplement 1 [file tvst-07-05-22_s01.pdf]

MAP SIMULATIONS WITH AN OBSERVER MODEL

As a first step in testing the multi-line adaptive perimetry (MAP) assessment, we developed a realistic observer model to examine theoretical changes in task efficiency and localization accuracy while exploring the parameter space of test features and plausible observer characteristics. The simulated observer had the following characteristics: 1) A retinal grid (e.g. visual field) with fixed location of underlying defect (e.g. metamorphopsia) represented as a two-dimensional circular region in a designated part of the visual field to serve as the "ground truth" for model evaluation (see Figure 1a), 2) an index of disease severity (IDS) to indicate the likelihood that a distortion will be selected (e.g. a FA) when the affected region happens to be sampled by the stimulus array (see Figure 1b), 3) stochastic cognitive perceptual motor (CPM) errors in terms of detecting the location of targets and providing a simulated pointing response to that location on the screen, and 4) a trait based generalized level of task accuracy (e.g. the hit rate) to represent the ability of the observer to detect targets and perform the overall task, which could vary by age or other characteristics of the observer cohort such as reduced visual field sensitivity.

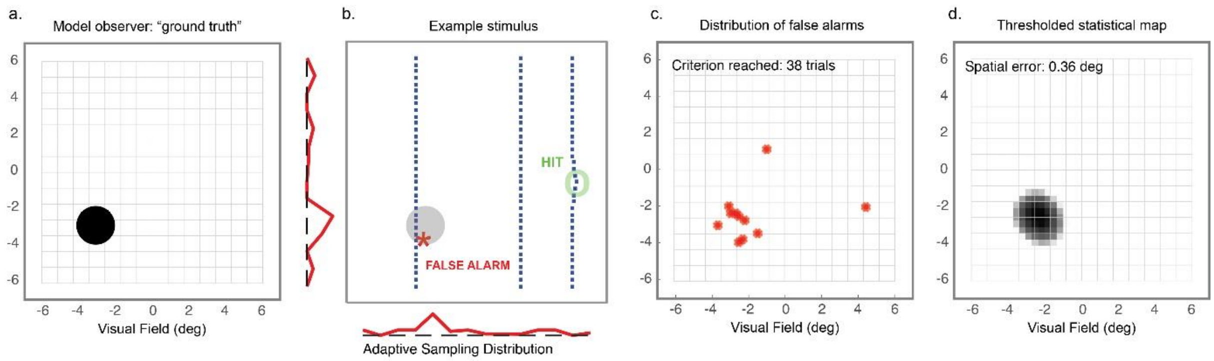

Figure 1. Illustration of observer model and simulation procedure. (a) The model observer has a retinal grid with a specified region of metamorphopsia to serve as the ground truth for model evaluation. (b) The model is presented with stimuli and responds to true targets (hits) and may

respond with a false alarm when a line crosses the metamorphopsia region. The red lines on the x and y axes illustrate the probability distributions for adaptive sampling following several simulated trials, showing that the probability of future sampling is increased for line locations with prior false alarms. (c) Criterion is reached and the simulated test is terminated when a sufficient number of false alarms have clustered in a region of space. A fewer number of trials needed to reach criterion indicates higher efficiency. (d) False alarms are analyzed and a thresholded statistical map is compared to the ground truth to determine localization accuracy.

These characteristics were embodied as parameters in the observer model, which we varied to explore a range of individual and population level characteristics in terms of disease status and performance ability. For instance, to simulate a healthier observer with a relatively intact CPM system and mild visual field deficits (e.g. more similar to characteristics of early-stage MD), we would specify a retinal grid with a minor deformation (distortion area = 1 deg diameter), low IDS (0.25 or 25% likelihood of perceiving distortion when the affected area is sampled), a small range of errors due to CPM factors (low sigma of error distribution), and a reasonable level of overall accuracy (90% hit rate). Alternatively, to simulate a less healthy observer with severe limitations to CPM and a larger visual disturbance (e.g. more similar to characteristics of late-stage MD), we would specify a major retinal deformation (distortion area = 3 deg diameter) with high IDS (0.75), high sigma for the CPM error distribution, and relatively lower accuracy (70% hit rate).

Method

Test Design Optimization

To derive an optimal test design according to simulated observer data, we manipulated two primary test features: (i) the number of lines presented each trial and (ii) the method of spatial sampling, for example, whether line locations were chosen with random uniformity in each trial or according to an adaptive algorithm that takes into account prior responses (Figure 1b). Test design optimization was achieved by simulating thousands of trials with various observer cohorts (defined by a particular set of observer model parameters), and identifying test features that maximized both test efficiency and spatial localization accuracy. Test efficiency was operationalized as the average number of trials it took to reach criterion-level confidence in detecting metamorphopsia, while accuracy was measured by the Euclidean distance error between the peak of estimated location of metamorphopsia and the ground-truth location of the model (i.e. the center of the circular region; see Figure 1a).

The overall simulation model contains several parameters that define observer characteristics, as well as features that represented test design characteristics; as a result, it would be impractical to search this entire parameter space. Instead we identified three practical sets of observer cohorts (Table 1) that spanned a reasonable range of model parameters. We also examined modifications of the test design by systematically incorporating different sets of test features. We specified a total of 8 unique test designs ranging from having 1 up to 4 lines presented per trial and with either adaptive or non-adaptive (random, uniform) spatial sampling (Table 1). The most basic case of this psychometric test resembles the PHP test in which a single line is presented each trial, a single response is allowed by the observer, and line locations are chosen randomly, or non-adaptively, from trial to trial (Loewenstein et al., 2003).

Adaptive Sampling Procedure

The sampling location of lines each trial were determined according to one of two schemes: 1) in the non-adaptive case locations were chosen randomly and independently from a uniform distribution of all possible locations, and 2) in the adaptive case, the probability of sampling a line location was dependent on prior responses, namely the relative frequency of previous FAs across space. The adaptive algorithm was initiated on trial 1 by sampling uniform locations. However as the test progressed, if a line was presented and there was no FA on it (e.g. correct rejection), then this was taken as marginal evidence that there was no metamorphopsia along that line and the probability of sampling that line in the future decreased according to parameter w1. On the other hand, if a line sample did result in a FA, then this was taken as evidence that there might be a metamorphopsia in a region crossed by that line, and the probability of sampling that line in the future increased according to parameter w2. In these simulations, w1=-0.02 and w2=0.1, so the relative influence of a FA on line sampling probability was five times greater than a correct rejection. Following each trial and the application of w1 and w2, the sum of the sampling distribution was normalized across all possible line locations to equal one to derive a probability distribution for sampling lines on subsequent trials. The main idea behind the adaptive algorithm was to increase the likelihood of sampling regions of the visual field that already had evidence for possible metamorphopsia. There is an advantage to sampling suspected regions more frequently as it increases the likelihood of a FA or missed target and evidence will accumulate faster if there is truly an underlying visual disturbance in that region.

**Observer Model Characteristics**				
**Simulated**	**Distortion area**	**Index of**	**Cogn-Percep-**	**Trait-based**

**Observer Cohort**	**size**	**Disease Severity**	**Motor error**	**Accuracy Rate**
Low severity	Minor (1 deg)	0.25	Small (1 deg)	90%
Mid severity	Fair (2 deg)	0.5	Medium (1.5 deg)	80%
High severity	Severe (3 deg)	0.75	Large (2 deg)	70%

**Test Design Characteristics**				
**Test Name**	**#Lines/trial**	**#Bumps/trial**	**#Responses/trial**	**Adaptive**
1Line	1	1	up to 2	No
1Line+Adaptive	1	1	up to 2	Yes
2Line	2	1 to 2	up to 3	No
2Line+Adaptive	2	1 to 2	up to 3	Yes
3Line	3	1 to 3	up to 4	No
3Line+Adaptive	3	1 to 3	up to 4	Yes
4Line	4	1 to 4	up to 5	No
4Line+Adaptive	4	1 to 4	up to 5	Yes

Table 1. Characteristics of model parameters for three simulated observer cohorts (top) and eight distinct test designs (bottom).

Simulation Procedure

The observer model is a non-deterministic model, so we performed 500 independent simulations to obtain a snapshot of average results for each condition (24 total conditions including 3 observer cohorts and 8 different test designs). Each simulation ran trial-by-trial in a manner similar to an actual human observer (as described in Figure 1), where a stimulus was generated with the designated number of lines (from 1 to 4) to span a portion of the visual field either vertically or horizontally (orientation determined by coin flip). A random number of targets, or bumps, (from 1 up to the total number of lines) were placed in random locations along the lines (with a constraint of up to 1 bump per line). The model observer knew the true location of each target and responded successfully to that location with a probability according to the model parameter for trait-based accuracy. After determining the classification of hits and misses to real targets, the model evaluated whether any portion of a line overlapped with the ground truth location of metamorphopsia within the model's "visual field" (Figure 1a-b). If so, then the model observer responded with a FA to that location with a probability equal to the model parameter specifying the index of disease severity (IDS).

For simplicity of model evaluation, the model observer always contained a single circular region designated as metamorphopsia that could vary in size (diameter). Before being recorded for analysis, the positions of model responses to targets and FAs were perturbed by a stochastic process to simulate the pooled influence of cognitive-perceptual-motor error. The error was represented as a vector from the target center, where the magnitude was approximated by a random

draw from a normal distribution with mean 0 and sigma equal to the model parameter specifying CPM error magnitude, and the radial direction was determined by a uniform random draw from 0 to 360 degrees. During the simulated task, we kept track of all locations in which a FA occurred (Figure 1c) for statistical analysis of FA clustering.

Analysis

Permutation Testing

Smoothed statistical maps of visual field integrity were computed following each trial by examining the probability associated with the spatial clustering of FAs (Figure 1c-d). The procedure for computing the map was as follows: 1) create an image of zeros (160 by 160) spanning + 8 deg of the visual field from center, where each pixel represents 1/10 deg, 2) replace a value of 1 for each FA location in the map, 3) smooth the image by convolving with a 2d Gaussian filter (sigma =10 pix, or 1 deg, and 4) threshold the map according to a statistical criterion.

To establish an appropriate criterion for evaluating statistical significance, we used a random permutation test to compute the distribution of maximum values expected by chance (e.g. the method of maximums) using the same analysis pipeline on an equivalent number of points chosen uniformly and randomly across visual space. Specifically, we simulated a thousand examples comprising n random point locations and stored the maximum value from the resulting smoothed map to create a null distribution of maximums. Maximum values of the simulated map will tend to be high only when there is clustering of points by chance; otherwise, with no clustering the maximum value of the smoothed map will tend to be lower. We used the 95th percentile of the null distribution of maximums as the threshold for evaluating statistical significance. For example, given that there are 10 FAs produced by the model observer, then regions in the smoothed map

that exceed the threshold criterion are by definition greater than the maximum value of 95% of smoothed maps derived from 10 locations chosen at random. The observer simulation was terminated when the maximum possible trials (240) was reached, or when the two following criteria were reached: 1) at least 8 total FA events had occurred (to make sure enough evidence had accumulated), and 2) the thresholded statistical map of visual field integrity must have a total area equal to or greater than the area of the simulated distortion region. Following termination, we recorded the number of trials it took to reach criteria (as a measure of efficiency; Figure 1c), and measured the Euclidean distance between the center of the "ground truth" region of the simulated observer and the peak location in the statistical map of visual field integrity (Figure 1d).

Results

Test Efficiency

Mean results of observer model simulations in terms of test efficiency are shown in Figure 2a, with each of three panels corresponding to a particular observer cohort (low, mid or high severity). Two significant trends emerge: 1) test efficiency increased as the number of lines per trial increased, as evidenced by the trend for fewer trials needed on average to reach statistical criteria, and 2) adaptive sampling added a further improvement to test efficiency in comparison to non-adaptive sampling, reflected by the observation that the green markers are consistently lower than the red markers across all conditions. There are also interesting differences among the observer cohorts in terms of test efficiency, in which fewer trials were needed on average to reach criteria for the mid-stage cohort in comparison to the early and late-stage cohorts. This is likely due to an interaction of the IDS parameter (likelihood of producing a false alarm) with the CPM parameter (the standard deviation of spatial localization errors). Since efficiency improvements

appear to saturate, or level off, at about 3 lines/trial, we focused quantitative comparisons on the 1-line versus 3-lines conditions. In the case of non-adaptive sampling, we found that about 63-64% fewer trials on average were needed to reach criterion in the 3-line condition across all three cohorts. For instance, in the most challenging case of detecting metamorphopsia in the less severe cohort (because of low IDS and therefore infrequent FAs), we found that it would take about 185 trials on average to reach criterion with only 1 line, whereas it would only take about 68 trials with 3 lines. In practical terms, this means that metamorphopsia may be detected with the same level of certainty, for example, in 10 minutes with 3 lines per trial, whereas it might take up to 30 minutes with only one line per trial, which is substantial when considering constraints of testing in a clinical setting.

For adaptive sampling we found that 49-57% fewer trials were needed for the adaptive case to reach criterion-level performance in identifying the underlying metamorphopsia across the three observer cohorts in comparison to the non-adaptive case. In general, better test efficiency translates to quicker assessment of the underlying disease state (or lack thereof), or it can produce a more reliable estimate in the same amount of time as a test with less efficiency. We surmise from these results that using 3 lines/trial could produce a near optimal balance between test efficiency and overall cognitive burden (e.g. the amount of visual attention and memory needed to encode multiple stimuli).

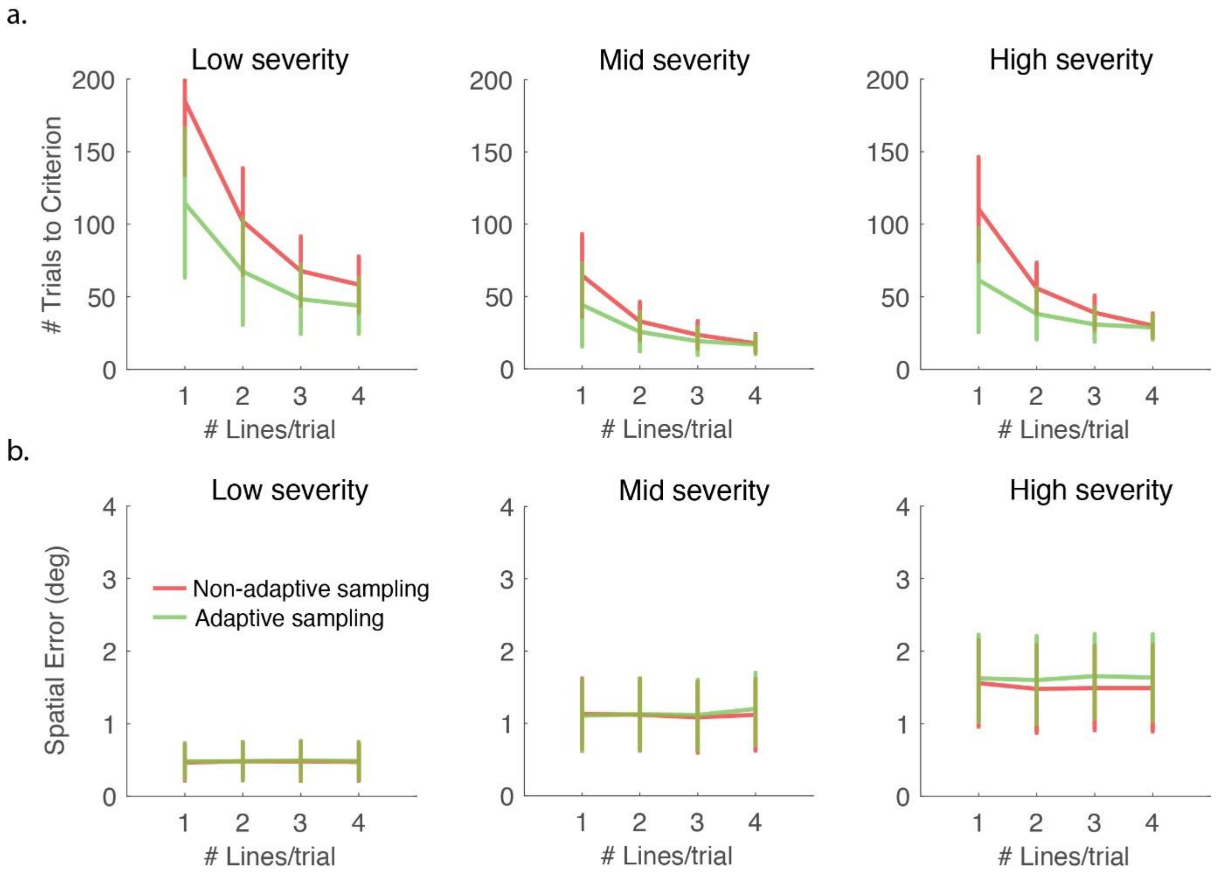

Figure 2. Results for Experiment 1 showing (a) efficiency and (b) localization accuracy for each type of test represented by the number of lines shown per trial (x axis) and whether there was adaptive sampling (green) or non-adaptive sampling (red). Each column of panels represents mean data for simulations of different observer cohorts including low severity (left), mid severity (middle) and high severity (right). Error bars represent standard error (SEM) across the 500 simulated observers.

Spatial Accuracy

Mean simulation results for spatial accuracy are shown in Figure 2b. In contrast to test efficiency, we found that the total number of lines/trial was not a factor in determining localization accuracy. Rather, spatial accuracy was influenced predominantly by the degree of cognitive-

perceptual-motor errors represented by the CPM parameter of the model. Simulated observers in the low, mid, and high severity cohorts had CPM error sigmas of 1, 1.5, and 2 deg, respectively, corresponding to increases in mean spatial error of 0.49, 1.11, and 1.55 deg. The accuracy of the MAP test in localizing metamorphopsia via analysis of FA clustering is therefore constrained mainly by individual trait-based features reflecting perceptual, cognitive and motor error in executing the task. This finding highlights the importance of effective task training and explanation prior to initializing the actual test, and making sure that participants can make responses to the screen comfortably and reliably to limit errors due to CPM factors.

Together, these simulation results reveal a substantial increase in time efficiency for MAP designs that employ multiple line stimuli per trial, thereby facilitating more behavioral responses per trial, and designs that employ adaptive sampling to hone-in on regions suspected of metamorphopsia. Since there is a reducing marginal benefit to including more than 3 lines, coupled with the cognitive difficulty of encoding and responding to multiple brief stimuli, we hypothesize that an ideal MAP design would have 3 lines per trial and adaptive sampling. We examine this hypothesis directly in Experiment 1 of the manuscript.
