# Supplementary material for: Multi-line Adaptive Perimetry (MAP): A New Procedure for Quantifying Visual Field Integrity for Rapid Assessment of Macular Diseases
Source: Transl Vis Sci Technol. 2018 Oct 16;7(5):28. doi: 10.1167/tvst.7.5.28 (PMC6192464; doi:10.1167/tvst.7.5.28)
Supplement: Supplement 2 [file tvst-07-05-22_s02.pdf]

Individual Case Studies

In the manuscript, we selected three patients (MD 5-7) as representative examples to discuss correspondence between anatomical fundus images and outcomes measures from MAP and MAIA. In Supplemental File 2, we include detailed case studies of the remaining patients enrolled in the study (MD1-MD4 and MD8).

Patient MD1

Patient MD1 is a 73 year old diagnosed with wet age-related macular degeneration (MD) in which there is a bilateral scotoma encompassing nearly the entire inferior macula (upper visual field) of eyes OD and OS (Figure 1). The boundary of the scotoma is highly visible in the fundus images, and the loss of visual sensitivity in this area is supported by MAIA microperimetry threshold measurements. Although the MAIA exam was obtained 13 months prior to the current experiment, MAP behavior responses showed a pattern highly consistent with MAIA. There was a strong tendency for missed targets in the inferior macula region and the resultant accuracy maps show very poor accuracy (0.0 – 0.2) for most retinal loci in this region, a result that was highly repeatable from test 1 to test 2. Interestingly, MAP FA distortion maps showed a highly significant clustering across the entire superior macula, suggesting a strong degree of visual distortion and metamorphopsia in the lower visual field for this patient. In summary, MAP accuracy and FA distortion maps showed quite strong repeatability for both eyes of MD1, and a strong correspondence with retinal fundus images and patterns of MAIA sensitivity thresholds.

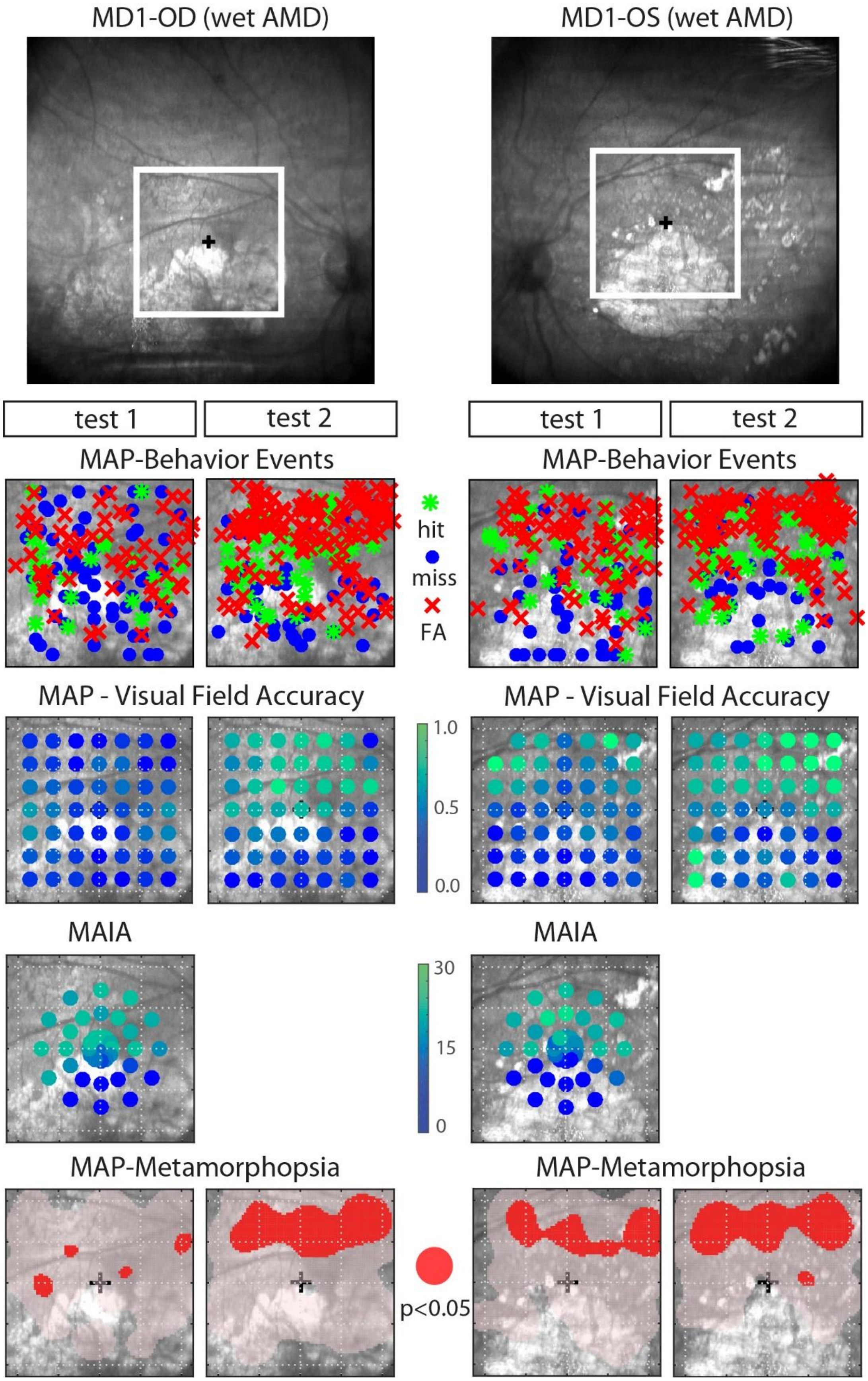
MD1-OD (wet AMD)
MD1-OS (wet AMD)
test 1
test 2
test 1
test 2
MAP-Behavior Events
MAP-Behavior Events
hit
miss
FA
MAP - Visual Field Accuracy
MAP - Visual Field Accuracy
1.0
0.5
0.0
MAIA
MAIA
30
15
0
MAP-Metamorphopsia
MAP-Metamorphopsia
p<0.05

Figure 1. (upper panel) Fundus image of OD and OS for patient MD1 diagnosed with wet AMD, with box identifying the central 14 deg of the visual field centered on the fovea. (lower panels) Test measurements from test 1 and test 2 associated with MAP and MAIA, overlaid on the corresponding location of the retina centered on the fovea. MAP data were scaled, inverted and then mirror reversed to transform from Cartesian coordinates to approximate retinal fundus image coordinates. The first lower panel shows MAP behavioral responses including False Alarms (red dots), Hits (green dots) and Misses (blue dots); the second lower panel shows MAP accuracy values resampled to the same resolution as the reference MAIA grid for direct comparison of measurements across retinal loci between the two tests. The third lower panel shows MAIA threshold sensitivity values. The fourth lower panel shows thresholded FA distortion maps of metamorphopsia, where regions marked red indicate a statistically significant degree of FA clustering.

Patient MD2

Patient MD2 is a 63 year old diagnosed with ocular histoplasmosis syndrome in which the fundus images show a clearly defined central scotoma in eye OD and diffuse retinal defects in eye OS (Figure 2). MAIA microperimetry thresholds were obtained 33 months prior to the current study, and showed a selective loss of visual sensitivity in the inferior macula of OD bounded by the anatomical scotoma; otherwise, sensitivity thresholds were in a normal range (>24 dB) in the superior macula of OD. In eye OS, MAIA showed relatively moderate sensitivity across the visual field but there was no region with a clear scotoma or complete loss of visual sensitivity. MAP behavior for OD showed a strong tendency for misses and FAs to cluster in the focal area circumscribed by the anatomical scotoma, leading to a repeatable loss of accuracy in

the same region represented by low MAIA thresholds. Also consistent with MAIA, accuracy was found to be moderate to high in the superior macula of OD. For eye OS, also similar to MAIA, MAP showed moderate performance across the entire visual field, with occasional misses that were well distributed and not significantly clustered in space. MAP FA distortion maps of OD showed a consistent clustering of FAs within and along the border of the anatomical scotoma in the inferior macula, which was repeatable from test 1 to test 2, suggesting a pattern of metamorphopsia and blurring of vision surrounding the scotoma. Eye OS showed overall less FAs, but there was a repeatable and statistically significant cluster approximately 3 deg nasal and 3 deg in the superior macula with a radius of 1-1.5 deg, suggesting metamorphopsia in this region of the visual field.

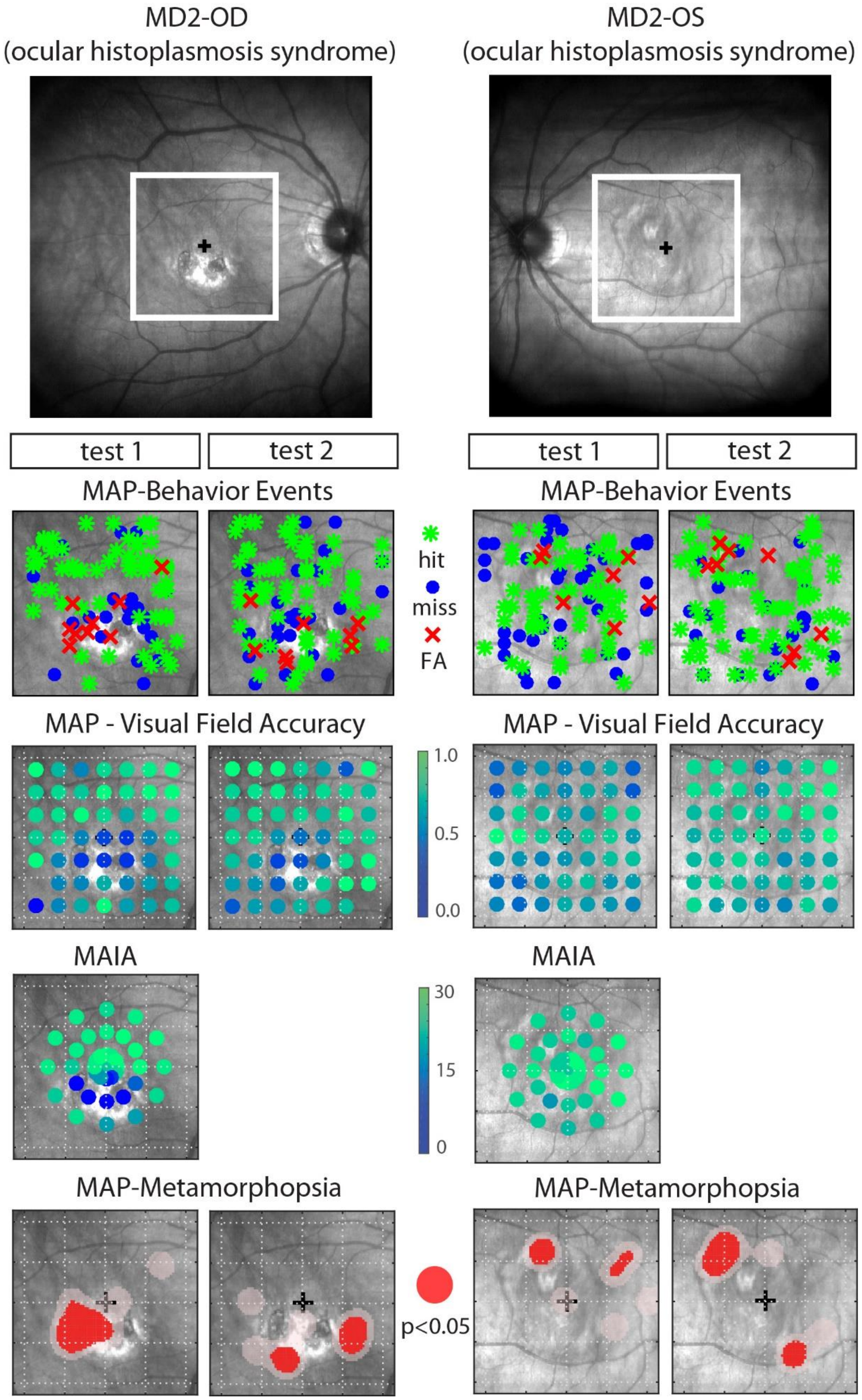
MD2-OD
(ocular histoplasmosis syndrome)
MD2-OS
(ocular histoplasmosis syndrome)
test 1
test 2
test 1
test 2
MAP-Behavior Events
MAP-Behavior Events
hit
miss
FA
MAP - Visual Field Accuracy
MAP - Visual Field Accuracy
1.0
0.5
0.0
MAIA
MAIA
30
15
0
MAP-Metamorphopsia
MAP-Metamorphopsia
p<0.05

Figure 2. (upper panel) Fundus image of OD and OS for patient MD2 diagnosed with ocular histoplasmosis syndrome. The figure elements are as described in Figure 1.

Patient MD3

Patient MD3 is a 50 year old diagnosed with a central scotoma of unknown etiology in which a MAIA exam was obtained 44 months prior to the current experiment for eye OD, but there was no MAIA exam available for OS. MAIA thresholds revealed a substantial loss of sensitivity across the entire nasal macular hemifield of OD, which did not correspond very closely with accuracy maps obtained by MAP (Figure 3). The discrepancy could be expected due to the length of time between the two exams (almost 4 years) and the progressive nature of macular degeneration. Accuracy maps did show a repeatable pattern of reduced accuracy in the superior macula, corresponding to lesser ability to detect targets in the lower visual field. Eye OS showed a similar and repeatable pattern of good accuracy in the inferior-nasal region, but lesser accuracy in the superior and temporal regions of the macula. MAP FA distortion maps showed a very similar pattern of metamorphopsia from test 1 to test 2 for both eye OD and OS. These clusters were scattered within 2-6 deg from the fovea in OD and from 1-4 deg from fovea in OS. While it is difficult to ascertain the correspondence between MAP and MAIA measurements due to the length of time between the two exams, these data show a strong degree of repeatability for MAP accuracy maps and FA distortion maps for each eye.

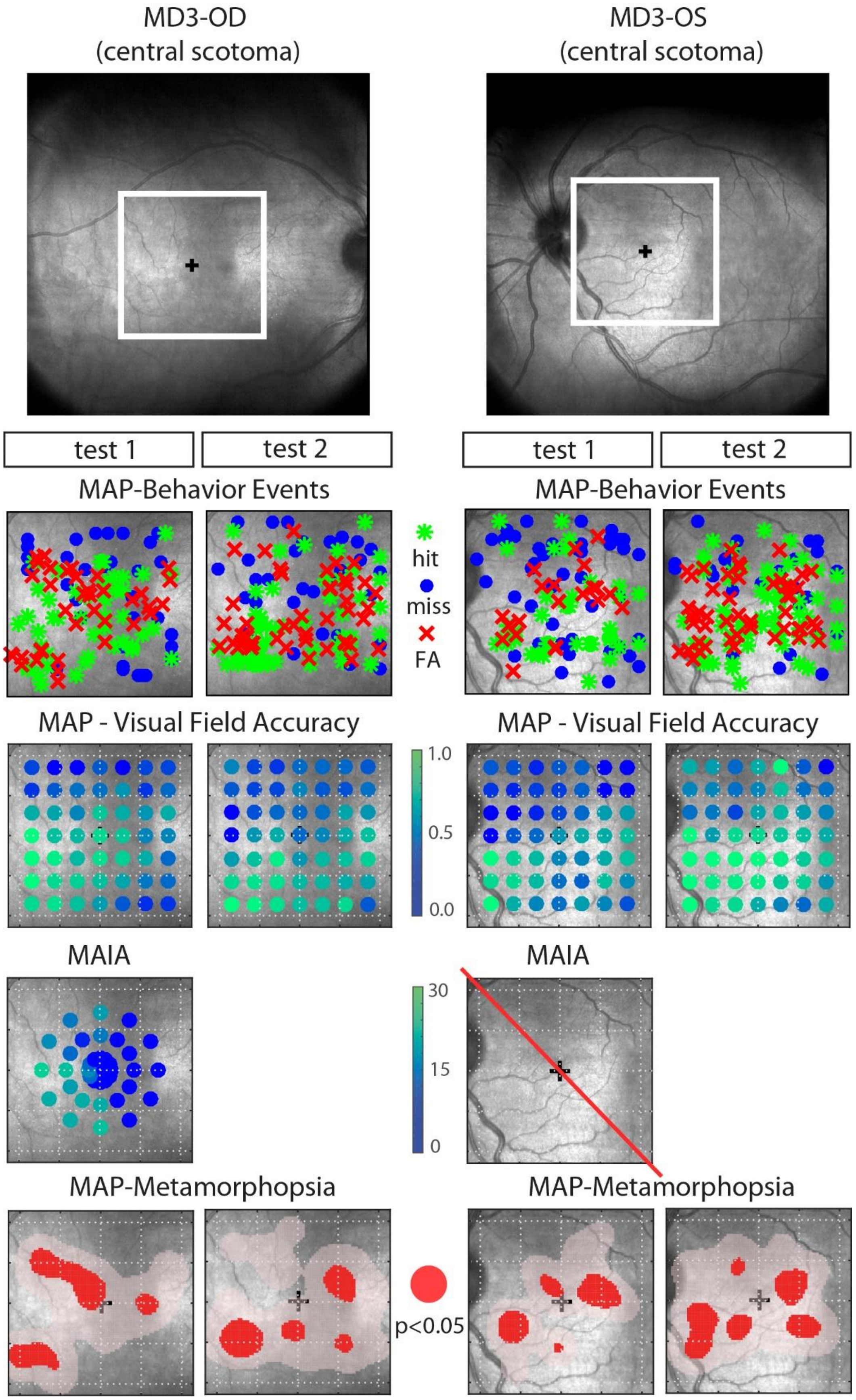
MD3-OD
(central scotoma)
MD3-OS
(central scotoma)
test 1
test 2
test 1
test 2
MAP-Behavior Events
MAP-Behavior Events
hit
miss
FA
MAP - Visual Field Accuracy
MAP - Visual Field Accuracy
1.0
0.5
0.0
MAIA
MAIA
30
15
0
MAP-Metamorphopsia
MAP-Metamorphopsia
p<0.05

Figure 3. (upper panel) Fundus image of OD and OS for patient MD3 diagnosed with central scotoma of unknown etiology. The figure elements are as described in Figure 1.

Patient MD4

Patient MD4 is a 50 year old diagnosed with diabetic retinopathy and central scotoma in eye OD encompassing the meridional temporal field of the macula, revealed by fundus photography and low MAIA thresholds in this region (Figure 4). For eye OS, MAIA thresholds showed a focal reduction of visual sensitivity inferior and adjacent to the fovea. MAP accuracy maps showed a general reduction of accuracy for OD in which most loci had accuracy less than 50%, but there was less focalization by comparison to MAIA. Eye OS showed reduced accuracy in the nasal and inferior fields of the macula. FA distortion maps showed a very repeatable pattern of localized clusters of metamorphopsia for both OS and OD between 1-4 deg from fovea. This degree of consistency of clustering of false alarm distortions is not likely to have occurred by chance, and strongly suggests scattered regions of perceptual distortion and metamorphopsia surrounding the fovea.

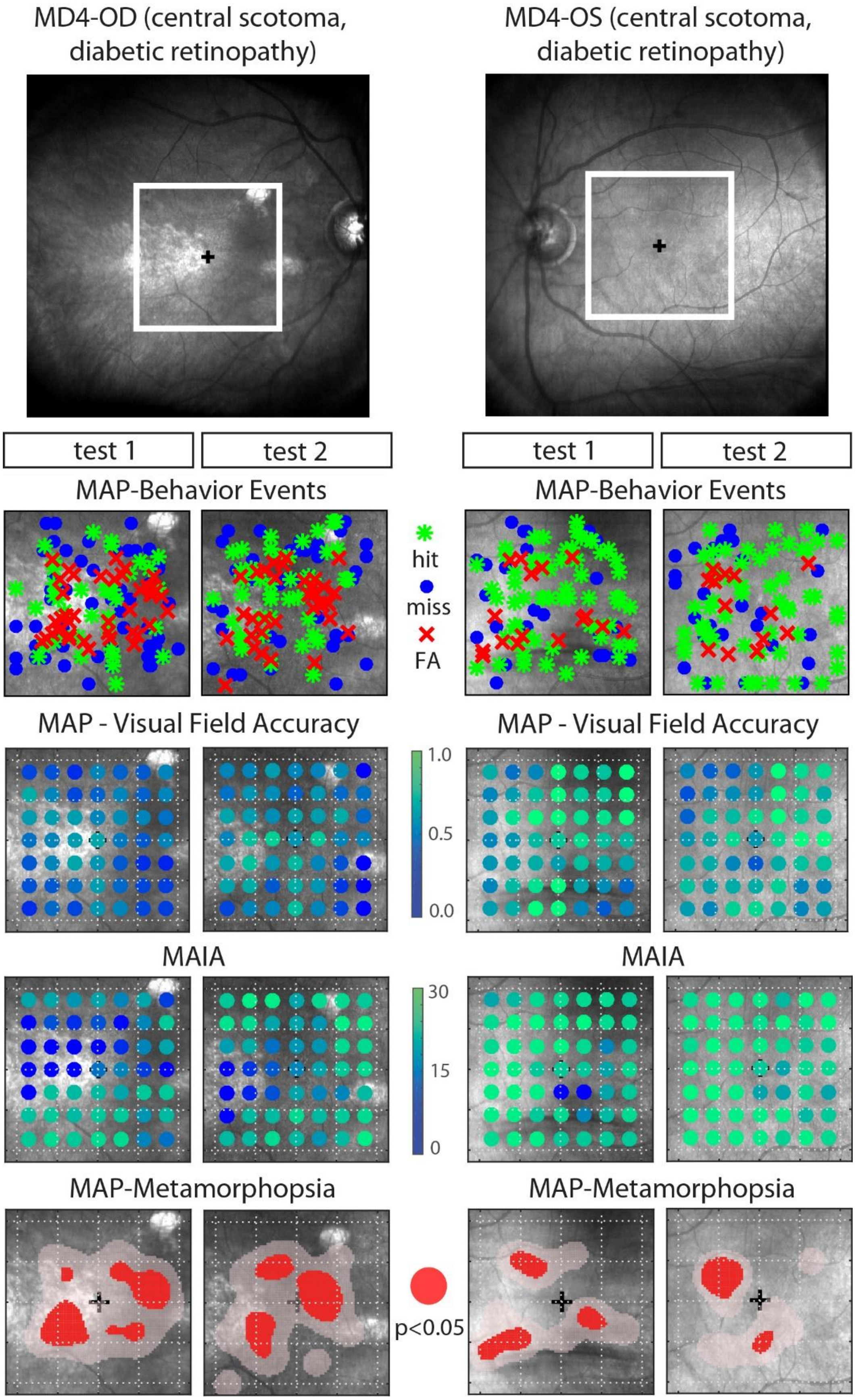
MD4-OD (central scotoma, diabetic retinopathy)
MD4-OS (central scotoma, diabetic retinopathy)
test 1
test 2
test 1
test 2
MAP-Behavior Events
MAP-Behavior Events
hit
miss
FA
MAP - Visual Field Accuracy
MAP - Visual Field Accuracy
1.0
0.5
0.0
MAIA
MAIA
30
15
0
MAP-Metamorphopsia
MAP-Metamorphopsia
p<0.05

Figure 4. (upper panel) Fundus image of OD and OS for patient MD4 diagnosed with diabetic retinopathy and central scotoma. The figure elements are as described in Figure 1.

Patient MD8

Patient MD8 is a 22 year old diagnosed with Stargardt's disease with bilateral scotoma in the inferior region of the macula, as indicated by anatomical defects in fundus images and a dramatic loss of visual sensitivity revealed by MAIA microperimetry (Figure 5). MAP accuracy maps also showed a corresponding reduction of accuracy in the inferior macula, corresponding to the upper visual field. This pattern of accuracy values was repeatable from test 1 and test 2 for both eye OD and OS. FA distortion maps for OD and OS showed a general pattern of metamorphopsia along the boundary of the scotoma in and around the fovea region, and just superior to the visible boundary of the scotoma. FA distortion maps were also very consistent from test 1 to test 2, highlighting the repeatability of accuracy and FA distortion maps derived from MAP responses.

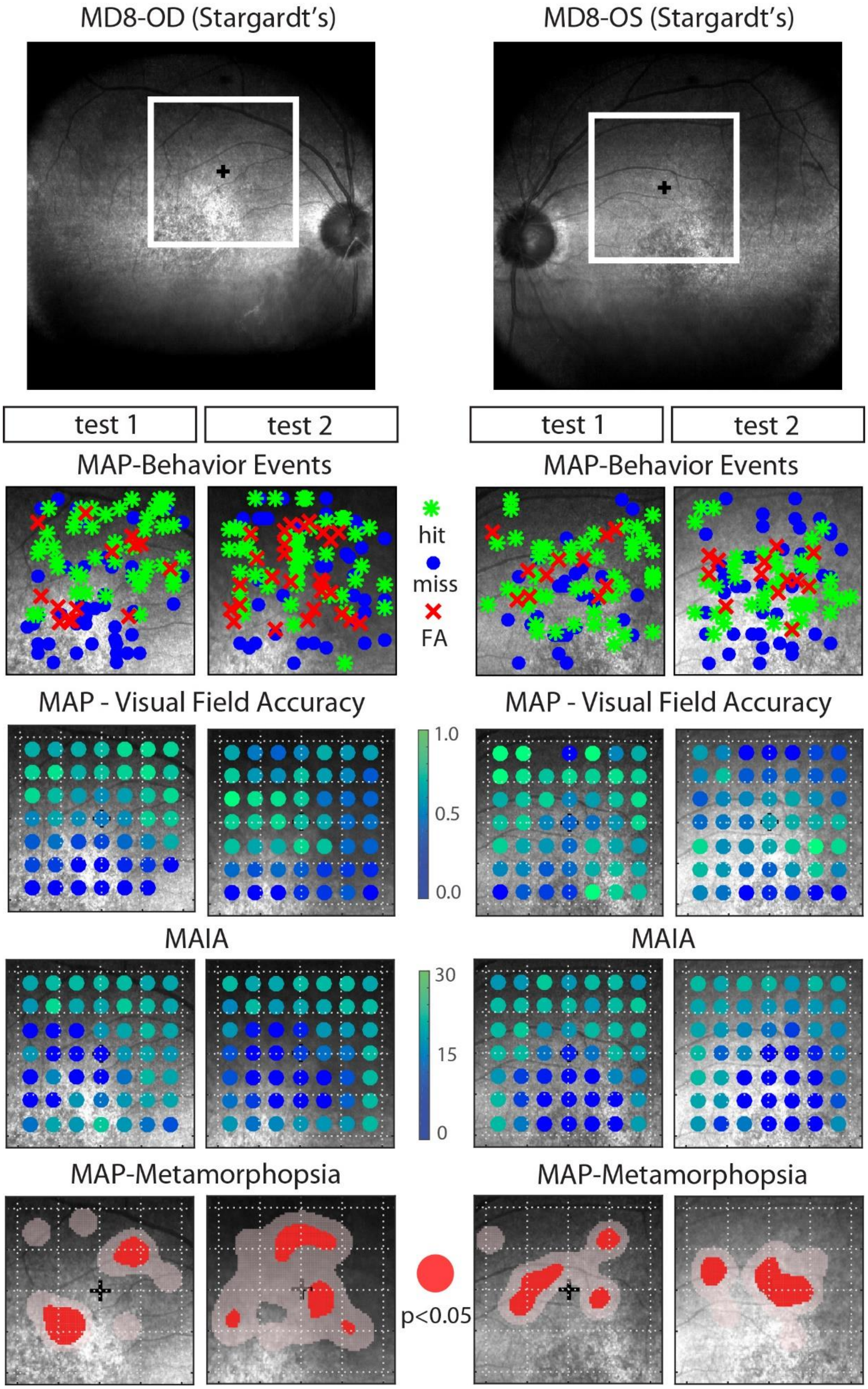
MD8-OD (Stargardt's)
MD8-OS (Stargardt's)
test 1
test 2
test 1
test 2
MAP-Behavior Events
MAP-Behavior Events
hit
miss
FA
MAP - Visual Field Accuracy
MAP - Visual Field Accuracy
1.0
0.5
0.0
MAIA
MAIA
30
15
0
MAP-Metamorphopsia
MAP-Metamorphopsia
p<0.05

Figure 5. (upper panel) Fundus image of OD and OS for patient MD8 diagnosed with Stargardt's. The figure elements are as described in Figure 1.
