# Supplementary material for: Multi-line Adaptive Perimetry (MAP): A New Procedure for Quantifying Visual Field Integrity for Rapid Assessment of Macular Diseases
Source: Transl Vis Sci Technol. 2018 Oct 16;7(5):28. doi: 10.1167/tvst.7.5.28 (PMC6192464; doi:10.1167/tvst.7.5.28)
Supplement: Supplement 3 [file tvst-07-05-22_s03.pdf]

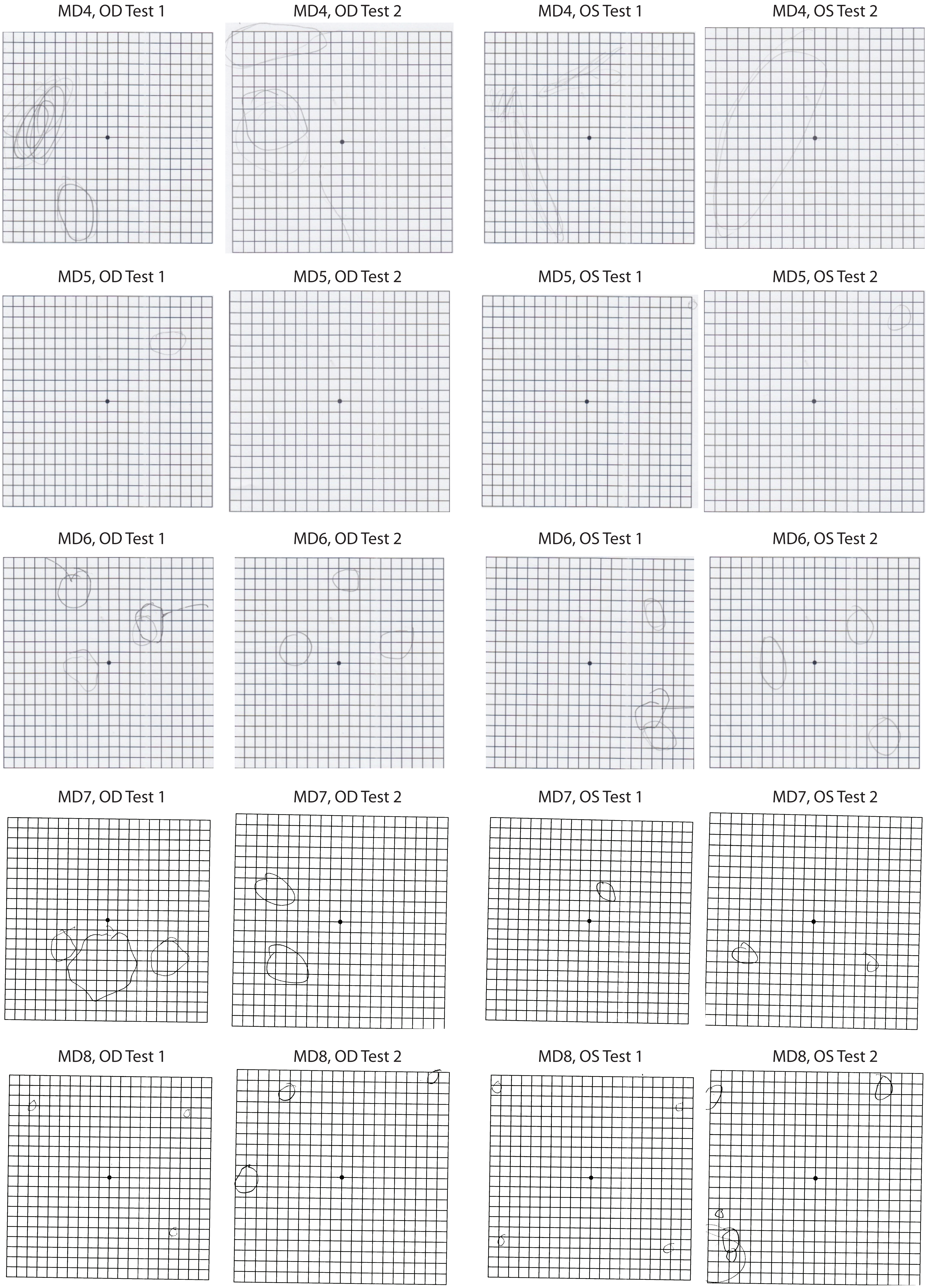

Supplementary Figure 1. Photocopies of Amsler grid markings of 5 patients (MD4-MD8) on test 1 and test 2 of the study. The demarcated regions were digitized (marked vs unmarked) manually (by author S.M.T) from these source images for analysis and comparison to MAP FA distortion maps.
